# Effects of caloric restriction on neuropathic pain, peripheral nerve degeneration and inflammation in normometabolic and autophagy defective prediabetic Ambra1 mice

**DOI:** 10.1371/journal.pone.0208596

**Published:** 2018-12-10

**Authors:** Roberto Coccurello, Francesca Nazio, Claudia Rossi, Federica De Angelis, Valentina Vacca, Giacomo Giacovazzo, Patrizia Procacci, Valerio Magnaghi, Domenico Ciavardelli, Sara Marinelli

**Affiliations:** 1 National Research Council–CNR, Institute of Cell Biology and Neurobiology, Rome, Italy; 2 IRCCS S. Lucia Foundation, Rome, Italy; 3 Department of Medical, Oral and Biotechnological Sciences, “G. d’Annunzio” University of Chieti-Pescara, Chieti, Italy; 4 Centro Scienze dell’Invecchiamento e Medicina Traslazionale—CeSI-MeT, Chieti, Italy; 5 Department of Biomedical Sciences for Health, Università degli Studi di Milano, Milan, Italy; 6 Department of Pharmacological and Biomolecular Sciences, Università degli Studi di Milano, Milan, Italy; 7 School of Human and Social Science, “Kore” University of Enna, Enna, Italy; University of Windsor, CANADA

## Abstract

There is a growing interest on the role of autophagy in diabetes pathophysiology, where development of neuropathy is one of the most frequent comorbidities. We have previously demonstrated that neuropathic pain after nerve damage is exacerbated in autophagy-defective heterozygous Ambra1 mice. Here, we show the existence of a prediabetic state in Ambra1 mice, characterized by hyperglycemia, intolerance to glucose and insulin resistance. Thus, we further investigate the hypothesis that prediabetes may account for the exacerbation of allodynia and chronic pain and that counteracting the autophagy deficit may relieve the neuropathic condition. We took advantage from caloric restriction (CR) able to exert a double action: a powerful increase of autophagy and a control on the metabolic status. We found that CR ameliorates neuropathy throughout anti-inflammatory and metabolic mechanisms both in Ambra1 and in WT animals subjected to nerve injury. Moreover, we discovered that nerve lesion represents, *per se*, a metabolic stressor and CR reinstates glucose homeostasis, insulin resistance, incomplete fatty acid oxidation and energy metabolism. As autophagy inducer, CR promotes and anticipates Schwann cell autophagy via AMP-activated protein kinase (AMPK) that facilitates remyelination in peripheral nerve. In summary, we provide new evidence for the role of autophagy in glucose metabolism and identify in energy depletion by dietary restriction a therapeutic approach in the fight against neuropathic pain.

## Introduction

Prediabetes reflects a metabolic alteration due to causes not completely known even if family history and genetics appear to play an important role. It is defined as a state of abnormal glucose homeostasis in which deficiency or resistance to insulin are distinctive features [1]. Prediabetes can prelude to type 2 diabetes or metabolic syndrome and it can be associated to different comorbidities such as neuropathy [2].

Derangement of glucose metabolism and hyperglycemia are regarded as key factors in the diabetes pathogenesis and comorbid neuropathy [3]. Interestingly, there is mounting evidence that autophagy, as key cell-protective mechanism, also plays a fundamental role in insulin secretion and β cells viability [4]. Basically, autophagy is a catabolic process of self-degradation for the preservation of cellular and mitochondrial homeostasis [5]. Autophagy is involved in the prevention of β cells death via its activity against protein aggregates that accumulates in β cells as consequence of hyperglycemia-induced oxidative stress [6–9]. Because of its fundamental role in degrading misfolded proteins, autophagy counteracts not only oxidative but also endoplasmic reticulum (ER) stress, which are both key elements in β cells toxicity and diabetes pathogenesis. Indeed, defective autophagy may increase ER stress that, in turn, is involved in insulin resistance [10]. Of note, hypoglycemic drugs for type 2 diabetes are β cells protective and autophagy inducers [11].

We recently demonstrated that pharmacological inhibition of autophagic activity, or defective autophagy in transgenic mice for the activating molecule in Beclin-1-regulated autophagy (Ambra1(+/gt), Ambra1 hereafter) [12], exacerbated allodynia response and persistence of neuropathic pain (NeP) [13]. Schwann cells (SCs) are essential for reparative process after peripheral nerve injury to control Wallerian degeneration (WD), which involves the progressive demyelination of peripheral nerves [14, 15]. Moreover, SCs are responsible for axons myelin ensheathment and paracrine trophic support to nerve [16]. When WD begins, SCs degrade and remove degenerated axons and myelin [14] throughout a process known as myelinophagy [4] (i.e. SCs autophagy) which contributes to prevent NeP chronification and facilitate nerve regeneration [13]. Nevertheless, the involvement of SCs in diabetic neuropathy and the role played by changes in energy metabolism and impairment of autophagy machinery is still mostly unknown. Interestingly, hyperglycemia has been observed to derange SCs function, induce apoptotic death [17] and de-myelination and de-differentiation of SCs into immature cells [18].

In view of accumulated evidence supporting the pathogenetic role played by autophagy in diabetes, we wondered whether autophagy-defective Ambra1 mice may show altered energy metabolism and diabetic-like signs, and whether a non-pharmacological enhancement of autophagy may revert or attenuate the exacerbation of NeP following nerve damage. For this purpose, the dual properties of caloric restriction (CR), as powerful autophagy mimetic and metabolic regulator, are here exploited. Energy depletion in the form of calorie intake restriction is a well characterized strategy to enhance autophagy, and promote health and longevity [19]. Hence, in the present study, we investigated the pathophysiology emerging from autophagy deficiency linked to metabolic dysregulation and peripheral neuropathy.

## Materials and methods

### Animals

Upon their arrival in the laboratory (at least 2 weeks before the experiments), animals were housed in standard transparent plastic cages, in groups of 4 per cage, lined with sawdust under a standard 12/12- hour light/dark cycle (7:00 am/7:00 pm), with food and water available ad libitum. Care and handling of the animals were in accordance with the guidelines of the Committee for Research and Ethical Issues of IASP (PAIN 1983, 16, 109–110) and with the European and Italian National law (DLGs n.26 del 04/03/2014, application of the European Communities Council Directive 2010/63/UE–Authorization n° DM 32/2014, Italian Ministry of Health) on the use of animal for research.

Wild-type (WT) CD1 male mice (Charles River Laboratories, Como, Italy) and heterozygous Ambra1 transgenic (CD1 background Ambra1+/gene trap–Ambra1+/gt) male mice 3 months old were used (obtained from Prof. F. Cecconi) [12]. Female mice were initially used to evaluate potential gender effects in the neuropathic phenotype of Ambra1 mice (S1 Fig). Not revealing significant differences in Ambra1 mice, all the experiments were performed in male mice.

All efforts were made to minimize animal suffering and to use only the number of animals necessary to produce reliable scientific data.

All the experiments were performed blind as for treatment group.

### Surgery

The procedure of monolateral chronic constriction injury (CCI) of sciatic nerve [20] induces mechanical allodynia and is a model of NeP.

It was performed under anesthesia (0.02 g/kg ketamine+ 5mg/Kg xylazine–Ketavet; Sigma-Aldrich USA; intraperitoneally -IP) as described elsewhere [21]. Briefly, the middle third of the sciatic nerve was exposed through a longitudinal skin incision, 3 ligatures were made with non-absorbable gut (5–0 chromic gut, Ethicon, Italy), tied around the sciatic nerve and the wound was then closed with 4–0 silk suture (Ethicon, Italy). Afterward, injured and uninjured hindpaw will be referred as ipsilateral and contralateral, respectively. Mice develop mechanical allodynia usually within 2–3 days. At the end of the experiments, mice were sacrificed, and the presence of the ligature was checked.

### Dietary restriction

In S1 Table are summarized all experimental groups, conditions, time-points and number of animals utilized.

Mice with body weight (BW) between 40 and 45 g were randomly attributed either to Standard diet (ST—ad libitum fed mice; n = 10/12 for each genotype) or to Caloric Restriction group (CR; n = 10/12 for each genotype) from the day of CCI (D0) up day 7 (D7) post-CCI.

To define the individual amount of feed ration, mice were isolated 15 days before the CCI and BW and food intake were recorded at approximately the same time (11:00 AM– 1:00 PM) for all mice. Food intake was determined daily by subtracting the weight of the food pellets remaining in the food hopper after the initial amount given (approximately 80 g).

During dietary restriction, mice in CR received weighed food pellets corresponding to the 40% less of their daily consumption in ad libitum condition. Pellets were dropped directly into each cage for easy access. Water was provided ad libitum.

### Mechanical allodynia

CCI-induced mechanical allodynia was tested by using a Dynamic Plantar Aesthesiometer (Model 37,400, Ugo Basile, Comerio, Italy) described elsewhere [21]. For habituation, mice were placed 30’ before test in the experimental room and in plastic cages with a wire net floor 5 min before the experiment. Starting from day 3 after CCI, withdrawal threshold was measured about one time at week (days 3, 7, 10, 14, 21, 28, 40, 45). The end point was defined by the total recovery from at least one experimental group. Each testing day, the withdrawal thresholds of ipsilateral and contralateral hindpaws were taken as the mean of 3 consecutive measurements per paw.

### Glycemia and triglycerides

Blood glucose and triglycerides (TGs) were measured using a Multicare Test Strips apparatus (Biochemical Systems International, Italy) by tail clipping. For glycemia, naïve animals were measured after overnight fasting while in baseline condition (BL, pre-CCI) and seven days after CCI (post-CCI) in normal feeding condition, both in ST and CR dietary regimen. For TGs, all experimental groups were measured in normal feeding condition. The same device was used to measure glycemia level in Glucose Tolerance Test (GTT) and Insulin Tolerance Test (ITT), here described.

### Insulin and glucagon serum levels

Insulin and glucagon were measured in the serum of control or CCI non-fasted mice using ELISA kits (RayBio Mouse Insulin Enzyme-linked immunosorbent assay (ELISA) Kit; RayBiotech Inc., Norcross, GA, USA and Quantikine ELISA Glucagon Immunoassay; USA & Canada R&D Systems, Inc) according to the manufacturer’s recommendations. Serum samples were harvested 24h, 3 days and 7 days after CCI. Specific antibody was coated onto the wells of the microtiter plates. The intensity of the signal was proportional to the concentration of insulin and glucagon present in the blood specimen and was read at 450 nm.

### Glucose tolerance test and insulin tolerance test

GTT was performed in naïve animals in WT (n = 10) and in A+/- (n = 11) mice. Plasma glucose at 0, 30’, 60’ e 120 min after intraperitoneal glucose administration (2g/kg) following overnight fasting (16h), was registered. For ITT (WT n = 8; A+/- n = 7), plasma glucose was recorded at 0, 30’, 60’ and 120 min after intraperitoneal insulin administration (1.5 U/Kg) following 5h fasting. Area under the curve (AUC) was calculated using trapezoidal rule.

### Inflammatory mediators

Levels of various cytokines/chemokines in the sera and sciatic nerves tissue lysates were analyzed using a mouse antibody array glass chip (RayBio Mouse Cytokine Antibody Array G series; RayBiotech Inc., Norcross, GA, USA) in WT mice in all experimental conditions. To obtain serum sample, blood was collected via beheading immediately following euthanasia, allowed to clot at room temperature for 30 min and then centrifuged at 3000 rpm for 15 min. Lysis buffer (Raybiotech, Inc) containing proteinase inhibitor (Sigma Aldrich) was added to sciatic nerve homogenates and 50 μg of each sample was added to the array. Incubation and washes were performed following the manufacturer’s instructions described in supplemental material. Fluorescence detection was performed using an Agilent G2564B microarray scanner (Agilent Technologies Italy) and analysis was performed using the array testing services from RayBiotech.

### Energy metabolism

Energy expenditure (EE), oxygen consumption (VO2) and respiratory quotient (RQ) were measured by an indirect calorimeter (IC) system (TSE PhenoMaster/LabMaster System, Germany) with a constant air flow of 0.35 L/min. Mice (N = 9–11 for each group) were adapted for 6 hour to the metabolic chamber, and VO2 was measured every 20 minutes in individual mice, starting at 7:00 PM and ending automatically after 48h (12h dark-light phase comparison). Room temperature was kept constant (22°±1°C). Formulae for parameters calculation are the following: RER = volume of CO2 produced/volume of O2 consumed, and is an index of substrate use. MR was calculated as EE = (3.815 + 1.232 x VCO2/VO2) x VO2, as provided by the TSE System. The MR and RER for each of the sample points were evaluated across the 48h of recording. Both MR and RER were also analyzed by considering animals’ resting conditions (values included between 0 and 3 activity counts). Locomotor activity was assessed during the IC by the number of infrared beams broken. Other details as described in a previous study [22, 23].

### Immunohistochemistry

Mice were sacrificed for immunofluorescence (IF) analysis of sciatic nerve (n = 3–5 for each group) before and after CCI. Sciatic nerves collection, samples preparation and double staining procedure are reported in [13]. Sections were first incubated overnight with primary antibodies and then with secondary antibodies for 2 hours at room temperature. For autophagy evaluation were used: anti-GFAP (mouse monoclonal, 1:100, Sigma-Aldrich or rabbit polyclonal, 1:100, Genemed) with anti-LC3 (mouse monoclonal, 1:100, nanotools) or anti P-mTOR (rabbit polyclonal, 1:100, CellSignaling) or P-AMPK (rabbit monoclonal, 1:100, CellSignaling).

For regenerative and proliferative processes were used: anti-GFAP (mouse monoclonal, 1:100, Sigma-Aldrich or rabbit polyclonal, 1:100, Genemed) with anti-GAP43 (mouse monoclonal, 1:100, Sigma Aldrich) or anti-Cdc2 (rabbit polyclonal, 1:100, Calbiochem), or anti-NF200 (rabbit polyclonal, 1:100, Sigma-Aldrich).

For myelin sheath analysis were used: anti-GFAP (as below) with anti-MPZ (chicken polyclonal, 1:100, Millipore) or anti-PMP22 (rabbit polyclonal, 1:100, Sigma-Aldrich).

Sections were incubated with secondary antibodies, a mix of goat anti-mouse fluorescein-conjugated (FITC, 1:100, Jackson Immuno Research) or goat anti-rabbit FITC (1:100, Santa Cruz Biotechnology) and goat anti-rabbit rhodamine-conjugated (TRITC, 1:100, Jackson Immuno Research) or donkey anti-chicken DyLight 549 (DYL, 1:100, Jackson Immuno Research) for 2 hours at room temperature.

Finally, the sections were washed in PBS and then stained with Hoechst-33258 (DAPI, 1:500, Sigma-Aldrich, USA).

### Confocal microscopy

Images of the immunostained sections were obtained by laser scanning confocal microscopy using a TCS SP5 microscope (Leica Microsystem, Germany). All analyses were performed in sequential scanning mode to rule out cross-bleeding between channels. High magnification (63X) images of sciatic nerve sections were operated by I.A.S. software (Delta Systems, Italy). Quantification was performed by using the ImageJ software (version 1.41, National Institutes of Health, USA).

Fluorescence of different proteins observed was quantified (at least 2 slices x n = 3 each group) by converting pixels in brightness values using the RGB (red, green and blue) as described in [24].

### Light microscopy

Sciatic nerves of perfused mice (2% paraformaldehyde and 2% glutaraldehyde in 0.1 M sodium cacodylate buffer (Sigma-Aldrich, USA) pH 7.3) were removed and post-fixed in 2% OsO4 (Sigma-Aldrich, USA), stained in 2% aqueous uranyl acetate and washed. Dehydrated samples were embedded in Epon-Araldite resin (Sigma-Aldrich, USA). Semi-thin (0.5 μm) sections, toluidine blue stained, were examined by light microscopy (LM) (Image Pro-Plus 6.0 software- magnification of 1500x).

### Protein isolation and immunoblotting

Sciatic nerves were homogenized in lysis buffer as described elsewhere [13] and mixed (n = 3 replicates for each WB), incubated on ice for 30 min and centrifuged at 13,000 g for 20 min. The total protein content of resulting supernatant was determined. Proteins were applied to SDS-PAGE and electroblotted on a PVDF membrane. Samples were incubated with the following primary antibodies: rabbit polyclonal anti-LC3, anti-AMPK, anti-pAMPK (T172), anti-pmTOR (S2448), anti-mTOR (Cell Signaling); rabbit polyclonal anti ß- actin (Sigma); mouse monoclonal anti-p62 (Santa Cruz); rabbit polyclonal anti-PMP22 (Millipore), rabbit polyclonal anti-MPZ (Abcam), rabbit polyclonal anti-pATG13 (Rockland), rabbit polyclonal Hsp90 (Santa Cruz).

All procedures for detection of Schwann cells autophagy in sciatic nerve follow the guidelines for autophagy monitoring [25].

### Metabolomics: Direct infusion mass spectrometry

Whole blood from each mouse was collected on filter paper card as dried blood spot (DBS). The determination of amino acids (AAs) and acylcarnitines (ACCs) was performed in DBS samples by DIMS analysis, as already reported [26–29]. Whole blood from each mouse was collected on filter paper card as dried blood spot (DBS), particularly suitable for small volume samples. The determination of amino acids (AAs) and acylcarnitines (ACCs) was performed in DBS samples by the addiction of isotopically labelled internal standards for each analyte of interest prior to the extraction, according to the principle of isotope dilution internal standardization. Filter paper disks were punched out from DBS samples and quality controls (QCs) using an automatic puncher, into a polypropylene microtitre plate. The diameter of the disk is approximately 3.2 mm (3–3.2 μL whole blood). 100 μL of the extraction solution containing internal standards were added to each well containing a filter paper disk. The internal standards as well as the extraction solution and the QCs were obtained from the NeoBase Non-derivatized MSMS Kit (Perkin Elmer Life and Analytical Sciences, Turku, Finland). Once covered, the plate was shaken in a thermo mixer (700 rpm, 45°C, 50 minutes). 75 μL from the contents of each well were transferred to a new microplate. The plate was placed in the autosampler for analysis. The QCs from the same kit were run in the same way of the real samples. The low and high blood spot QCs from the kit were run in replicate in each plate, before and after the real samples. The direct infusion mass spectrometry (DIMS) analysis for the evaluation of metabolite profile in DBS samples was performed using a Liquid Chromatography Tandem Quadrupole Mass Spectrometry LC/MS/MS system (Alliance HT 2795 HPLC Separation Module coupled to a Quattro Ultima Pt ESI, Waters Corp., Manchester, UK). The instrument operated in positive electrospray ionization, with multiple reaction monitoring (MRM) as acquisition mode, using MassLynx V4.0 Software (Waters Corp.) with auto data processing by NeoLynx (Waters Corp.). Autosampler injections of 30 μL were made into the ion source directly by a narrow peek tube. The total run time was 1.8 minute, injection-to-injection. The mass spectrometer ionization source settings were optimized for maximum ion yields for each analyte. Capillary voltage was 3.25 kV, source temperature was 120°C, desolvation temperature was 350°C and the collision cell gas pressure was 3–3.5 e-3mbar Argon.

The list of analyzed metabolites and a description of abbreviations as used in text are available in S3 Table.

### Experimental design and statistical analysis

Details regarding all experimental groups, conditions, time-points and number of animals are condensed in S1 Table.

Data were expressed as mean ± standard error of the mean. Complete results of the statistical analyses and exact p-values are reported in the results section and in S2 and S4 Table for metabolomics. Depending on data, statistical analysis was performed either by unpaired t test, 1-way analysis of variance (ANOVA) or 2-way ANOVA for repeated measures while for small samples (N<5 animals) and groups >3, non-parametric analysis was performed by Kruskall-Wallis.

Tukey–Kramer test has been used for post-hoc analysis in multiple comparison or t-Test for single comparison.

Variable Importance in Projection (VIP) scores was calculated by estimating the importance of each variable used in the PLS-DA model [30]. Baseline differences between WT and Ambra1 mice, the effects of CCI on whole blood AAs, ACCs, and sums of short-chain, odd-chain, 3-Hydroxy/diCarboxy, medium and long-chain, and long-chain ACCs in the same mouse strains were assessed by two-factor mixed design ANOVA followed by Fisher post-hoc test, using the general linear model (GLM) approach. Genotype and CCI were the independent factors.

The effect of CR on AAs, ACCs, and sums of 3-Hydroxy/diCarboxy, medium, and long-chain ACCs in post-CCI WT and post-CCI Ambra1 mice were assessed by two-factor ANOVA followed by Fisher post-hoc test with genotype and diet regimen as independent factors.

When the assumption of the homogeneity of the variance was rejected by Levene test, the aligned rank transformation (ART) of data was performed.

Unsupervised hierarchical cluster analysis, supervised Partial Least Square Discriminant Analysis (PLSDA), and Receiver Operating Characteristic (ROC) analysis were performed using MetaboAnalyst statistical analysis module [30] using the default settings.

The quality of the PLS-DA model was expressed by R2 and Q2 parameters, representing the explained variance and the predictive capability of the model, respectively.

A variable with a VIP score higher than one is considered important in a given model. Mean centering, unit variance scaling, and log transformation of metabolite concentrations were performed before multivariate data analysis.

## Results

### Ambra1 autophagy-defective mice show metabolic dyshomeostasis and prediabetes hallmarks

**Fig 1:**
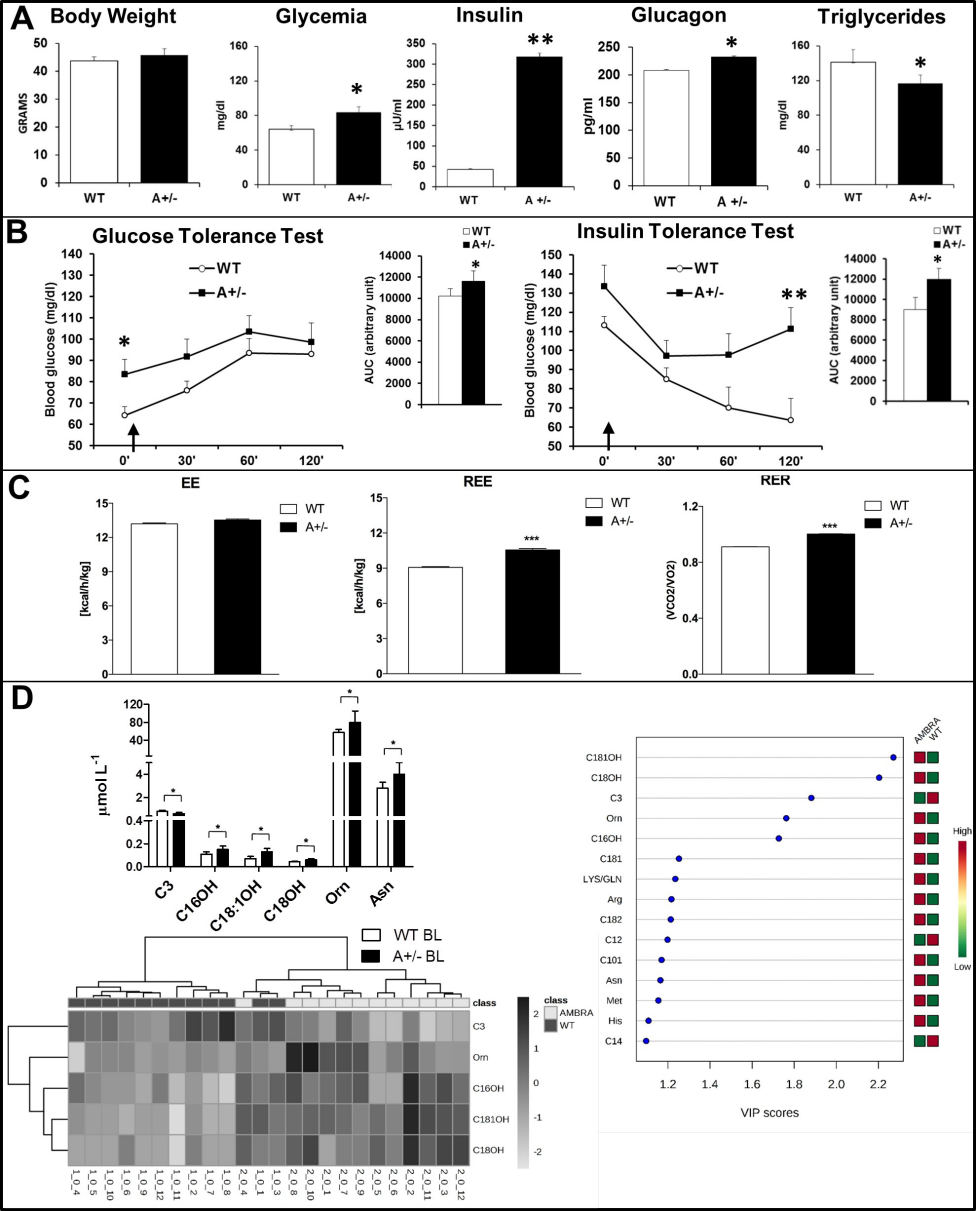
Metabolic phenotype of wild type and Ambra1 mice in baseline condition. (**A**) Body weight, Glycemia measured after overnight fasting, glucagon, insulin and triglycerides plasma levels in BL condition, showing significant differences between the two genotypes. (**B**) Glucose Tolerance Test: plasma glucose at 0 and 30’, 60’ and 120 min after intraperitoneal glucose administration (2g/kg, arrow) following overnight fasting. AUC shows significant increase (around 12%) in Ambra1 mice. ANOVA for repeated measures revealed significant effects for Time (p<0.0001) and for Time x Genotype interaction (p<0.05). Insulin Tolerance Test: plasma glucose at 0 and 30’, 60’ and 120 min after intraperitoneal insulin administration (1.5 U/Kg, arrow) following 5 hours fasting. AUC shows a significant increase (+32%) in Ambra1 mice. ANOVA for repeated measures revealed significant effects for Genotype (p<0.05) and Time (p<0.0001). (**C**) Energy metabolism measured by continuous 48-h recording of energy expenditure (EE), total resting EE (REE) and respiratory exchange ratio (RER), assessed via the indirect calorimetry. Heat is expressed as Kcal emitted per hour (h)/Kg. (**D**) Whole blood amino acid (AA) and acylcarnitine (ACC) profiling of wild type (WT BL) and Ambra1 (A+/- BL). Variable importance on projection (VIP) plot: VIP scores >1 indicate a high relevance for the selected AAs and ACCs in the predictive model. Heatmap visualization of unsupervised hierarchical clustering analysis based on whole blood concentrations of C3, C16OH, C18:1OH, C18OH, and Orn in the study groups. Each bar represents a metabolite coded in accordance with its concentration expressed with a normalized scale ranging from light grey (low level) to dark grey (high level). Rows depict analysed AAs and ACCs, columns indicate samples * p<0.05 and ** p<0.001 vs WT.

The analysis of metabolic phenotype discloses the existence of a prediabetic condition in Ambra1 mice. Body weight (BW) did not differ between the two genotypes (slightly increased in Ambra1 mice), but Ambra1 mice resulted hyperglycemic after overnight fasting (t_19_ = 2,272, p .034) and hyperinsulinemic (t_4_ = -31,05, p < .0001) and hyperglucagonemic (t_4_ = -9,834, p .0006), in non-fasting conditions (Fig 1A). Triglycerides plasma levels were lower in non-fasted Ambra1mice (t_30_ = -2,217, p .034; Fig 1A*)* that also showed significantly higher glycemia levels after 16h fasting (time 0’) and an increase of glycemic levels after glucose loading (GTT and AUC; main effect—time F_3,19_ = 16,43 p < .0001; t_19_ = 2,272, p .034; Fig 1B). In ITT a pattern of insulin resistance with significant higher blood glucose levels was found in 5 hours fasted mice (main treatment effect F_1,15_ = 6,61 p .021; main effect–time F_3,15_ = 22,02 p< .0001; 60’: t_15_ = 2,15 p .048; 120’: t_15_ = 2,57 p .021; Fig 1B).

To further understand whole-body energy metabolism, we used the indirect calorimetry (IC) analysis to investigate energy expenditure (EE), resting EE (REE) and nutrient substrate oxidation (respiratory exchange ratio, RER) in autophagy-defective mice. Fig 1C shows that in BL condition no differences are observable in EE between WT and Ambra1 mice; by contrast, higher REE was showed by Ambra1 mice (t_36_ = 9.28, p< .0001). Moreover, the increase of RER demonstrates that substrate oxidation, (i.e. fat oxidation), is reduced in Ambra1 mice (t_36_ = 14.01, p< .0001; Fig 1C).

Next, we investigated blood alterations in AAs and ACCs, as indirect markers of fatty acid and protein catabolism, to identify a panel of biomarkers as potential metabolic signature [31] of Ambra1 mice (Fig 1D). In BL, Ambra1 mice showed higher levels of long-chain 3-hydroxy ACCs (C16OH, C18:1OH, C18OH), AAs ornithine (Orn) and asparagine (Asn), and lower levels of C3 (propionylcarnitine) (see also S1 Table,S2 Fig for details and statistics). Supervised PLSDA showed that AAs and ACCs metabolic signatures discriminate between WT and Ambra1 mice (R2 = 0.91, Q2 = 0.75; S2 Fig) indicating that C3, C16OH, C18:1OH, C18OH, and Orn are the most important variables contributing to the separation between WT and Ambra1 mice in BL condition. Unsupervised hierarchical cluster analysis further highlights the importance of these five metabolites for genotype discrimination, providing a sensitivity of 100% and a specificity of 83.3%.

### Caloric restriction relieves neuropathic pain, nerve damage and Ambra1-associated metabolic alterations

**Fig 2:**
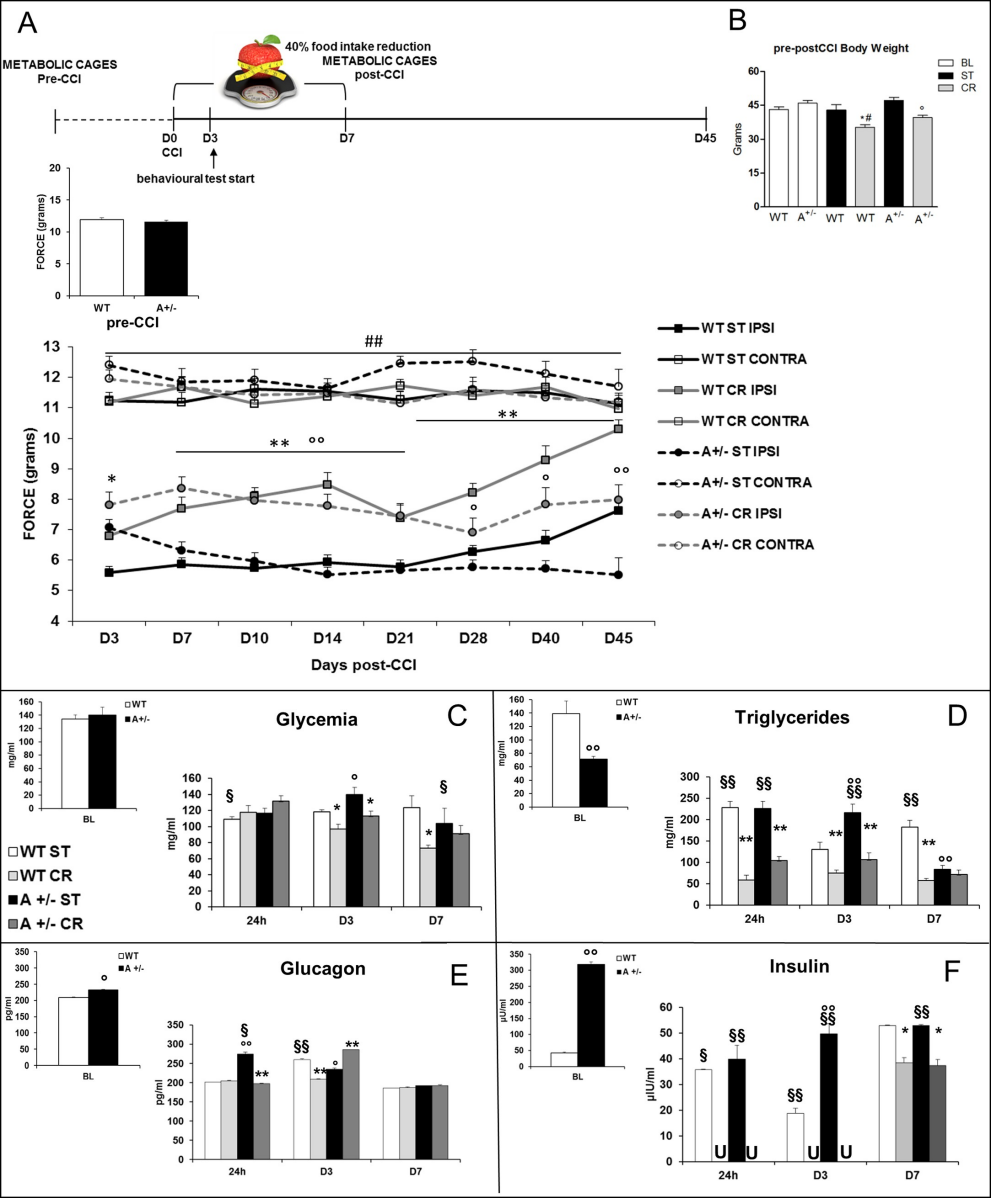
Metabolic changes associated to NeP and CR promote recovery. (**A**) The experimental timeline is schematically represented. Mechanical allodynia measurement before CCI (pre-CCI) shows no differences between WT and A+/- mice. Effects induced by CR or ST diet on mechanical allodynia. Repeated measures ANOVA evidenced: a diet effect (P< 0.0001) and an interaction between factors (genotype, diet and time–days postCCI; P<0.0001). From D3 to D45 *P<0.05 or **P<0.0001 WT CR ipsilateral (IPSI) vs WT ST IPSI hindpaw and° p<0.05 or°°P<0.0001 Ambra1 mice in CR diet (A+/- CR IPSI) vs A+/- ST IPSI. Only WT CR mice reached the contralateral (CONTRA) hindpaw (D45). (**B**) Body weight (g) changes at baseline (BL) and day 7 postCCI in ST or CR conditions. (**C**) Glycemia (**D**) triglycerides, (**E**) glucagon and (**F**) insulin plasma levels at BL and at different time points from ligature (24h, D3, D7). (U = undetectable, insulin values decrease under detection threshold (5 μU/l);°P<0.05°°P<0.001 vs WT; *P<0.05 **P<0,001 vs ST diet; §P<0.05 §§P<0,001 vs BL).

Mechanical allodynia was measured before mice underwent CCI (pre-CCI), and no differences were revealed between genotypes (Fig 2A). Mice that underwent CCI of sciatic nerve develop neuropathic pain in the hindpaw ipsilateral (IPSI) to the lesion, as showed in WT mice in ST condition (WT ST CONTRA vs WT ST IPSI Fig 2A). No significant differences were observed between allodynia level in contralateral hindpaw and measurement in basal condition (pre-CCI). Hence, contralateral hindpaw withdrawal threshold was considered as control level. We previously demonstrated [13] that Ambra1 mice subjected to CCI never recovered from neuropathy, as here confirmed (Fig 2A). When subjected to CR, both WT and Ambra1 mice reduced allodynia response (main treatment effect F_7,82_ = 264,04 p < .0001; interaction treatment*time F_49,574_ = 3,517 p < .0001 Fig 2A). WT totally recovered from neuropathy thus approximating the mechanical threshold showed by the contralateral hindpaw (Fig 2A, D45 WT CR CONTRA vs WT CR IPSI t_22_ = 1,6 p .123). Ambra1 mice showed a parallel but not identical reduction of allodynia significantly decreasing pain (A+/- CR vs A+/- ST; D7-D21/D45 p < .0001 Tukey HSD; D28-D40 p < .05 Tukey HSD).

By studying the metabolic changes induced by CR regimen, we unexpectedly discovered that CCI had *per se* a metabolic impact. Nerve injury produced different effects on the two genotypes depending on the exposure to CR. Indeed, robust changes of glycemia, triglycerides, insulin and glucagon were found during neuropathic pain development and CR regimen (Fig 2C–2F). As expected BW was lower in mice subjected to CR while CCI did not affect BW (Fig 2B).

In non-fasted animals glycemia (main treatment effect F_1,1_ = 8,37 p .009; main effect–time F_2,20_ = 6,31 p .004; treatment*time F_2,2_ = 6,3 p .004) was altered by the neuropathy induction, decreasing 24h after CCI in WT ST mice (WT ST vs WT BL; p < .05 Tukey HSD) and at 7 days in Ambra1 ST mice (A+/- ST vs A+/- BL, p < .05 Tukey HSD) as compared to the BL condition. CR was able to change glycemia, and in WT mice a lower glycemia was observable at D3 and D7 (WT CR vs WT ST, p < .05 Tukey HSD; Fig 2C) and at D3 in Ambra1 mice (A+/- CR vs A+/- ST, p < .05 Tukey HSD; Fig 2C).

Also triglycerides levels were affected by nerve lesion both in WT (24h and D7; WT ST vs WT BL p < .0001 Tukey HSD, Fig 2D) and in Ambra1 mice (24h and D3; A+/- ST vs A+/- BL, p < .0001 Tukey HSD), while CR decreased triglycerides in both genotypes at all time points (WT CR vs WT ST and A+/- CR vs A+/- ST; p < .0001 Tukey HSD) except than at D7 in Ambra1 mice (main treatment effect F_1,1_ = 223,1 p < .0001; interaction main treatment effect*genotype F_1,20_ = ; 6,93 p < .015; main effect–time F_2,20_ = 15,16 p < .0001; interaction genotype*time F_2,2_ = 12,62 p < .000; interaction main treatment effect*time F_2,2_ = 8,02 p .0012; interaction main treatment effect*time*genotype F_2,40_ = 8,74 p .0007, Fig 2D).

Moreover, CCI induced changes in glucagon levels, and hyperglucagonemia was observed in WT ST (D3) and Ambra1 ST mice (24h) (H_14_ = 43,262 p < .0001; WT ST vs WT BL t_4_ = 26,946 p < .0001; A+/- ST vs A+/- BL t_4_ = 8,365 p .0011; Fig 2E). CR counteracted hyperglucagonemia at 24h in Ambra1 mice and D3 in WT, reporting the level to basal value but induced a strong increase at D3 in Ambra1 mice (A+/- CR vs A+/- ST; t_4_ = -9,248 p .0008).

As for the other metabolic parameters, CCI produced also changes in insulin levels which were highly susceptible to CR (H_9_ = 25,09 p .0029; Fig 2F). A decrease was observed in WT ST mice at 24h (WT ST vs WT BL; t_4_ = -3,27 p .03), D3 (t_4_ = -8,38 p .0011) and D7 (t_4_ = -5,22 p .0064); as well as in Ambra1 mice (A+/- ST vs A+/- BL t_4_ = -27,26 (24h), -28,54 (D3), -30,78 (D7) p < .0001; Fig 2F), which were completely downregulated by CR at 24h and D3 in both genotypes. Conversely, at D7 we found a partial recover of insulin levels in Ambra1 CR mice, which showed values comparable to those observed before CCI in normoglycemic WT mice (Fig 2F).

**Fig 3:**
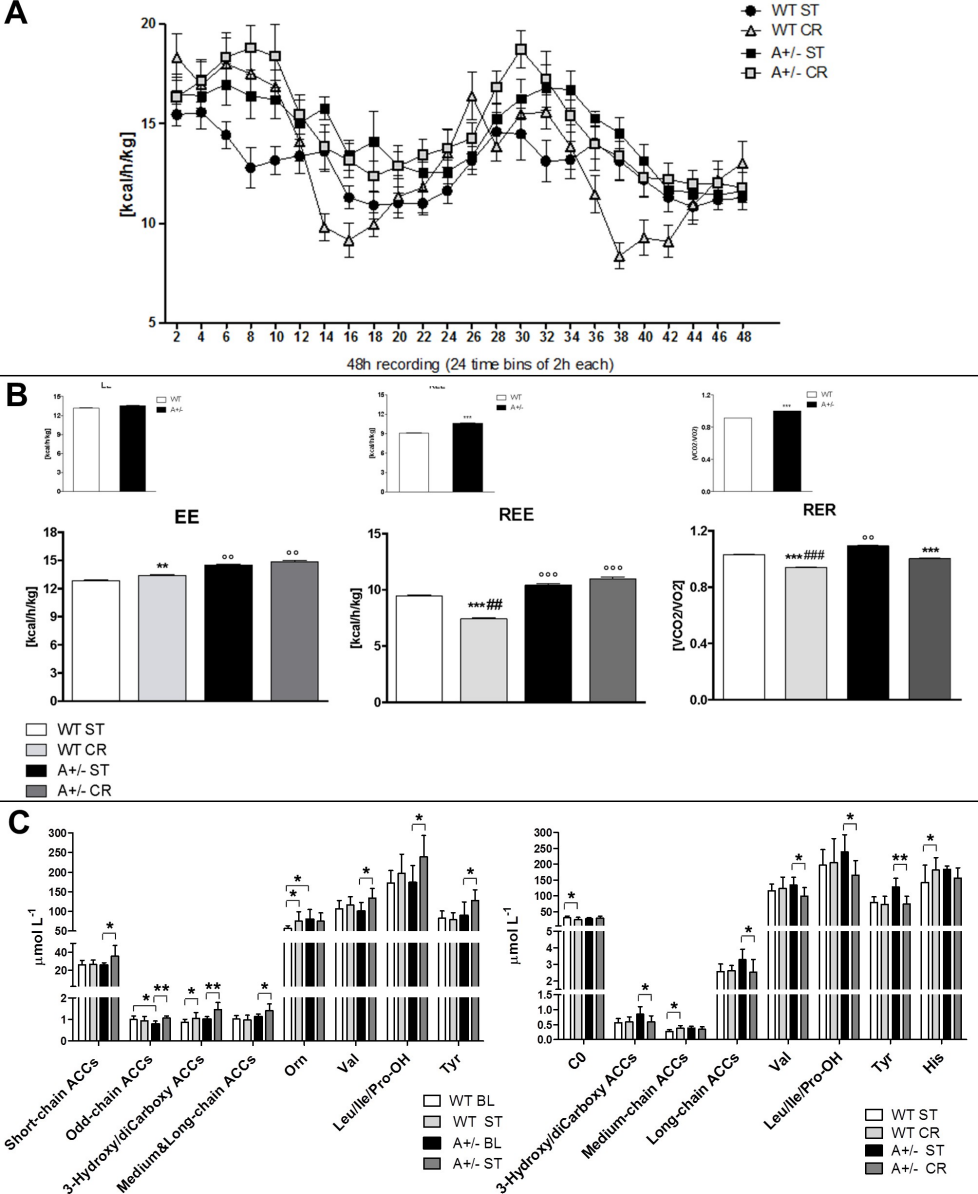
**Energy and metabolic profile of WT and Ambra1 (A**) Continuous 48-h recording energy expenditure (EE) in WT (ST and CR) and A+/- (ST and CR) mice assessed via the indirect calorimetry at D7 postCCI. Heat is expressed as Kcal emitted per hour (h)/Kg. (**B**) Mean of 48-h whole EE, total resting EE (REE) and respiratory exchange ratio (RER) in postCCI phase. (°P<0.05°°P<0.001 vs WT; *P<0.05 **P<0,001 vs ST; # P<0.05 ## P<0.001 vs A+/-). The small inlets above each main panel depict EE, REE and RER before in BL conditions as in Fig 1 (panel B). (**C**) Whole blood amino acid (AA) and acylcarnitine (ACC) profiling of WT BL and A+/- BL mice vs WT and Ambra1 mice 7 days after CCI in ST dietary regimen (WT ST and A+/- ST), and of WT ST and A+/- ST mice vs WT and Ambra1 mice under CR (WT CR and A+/- CR). * P<0.05, ** P<0.001.

The impact of CCI and CR on energy metabolism was reassessed by IC analysis, which showed changes in EE, REE and RER (Fig 3A and 3B*)*. We observed an increase of EE in WT mice that underwent CR (main treatment effect, F_(3,560)_ = 21.71, p< .0001; WT CR vs WT ST, p< .001 Tukey HSD), and a further increase of EE in Ambra1 mice as compared to WT groups (Ambra1 ST vs WT CR p< .001 and Ambra1 CR vs WT CR p < .001, Tukey HSD; Fig 3B). The same pattern of increase in Ambra1 mice was observed by considering only the volume of O2 consumed (ml/h) (S3 Fig). REE decreased in WT mice that underwent CR (main treatment effect, F_(3,472)_ = 151.3, p< .0001; WT CR vs WT ST, p < .0001 Tukey HSD), while the increase of EE observed in Ambra1 mice was also present in resting condition (Ambra1 ST and Ambra1 CR vs WT p < .0001, Tukey HSD; Fig 3B). RER was reduced in WT mice that underwent CR as well as in Ambra1 CR mice (main treatment effect, F_(3,560)_ = 583.3, p< .0001; WT CR vs WT ST and Ambra1 CR vs Ambra1 ST, p< .0001 Tukey HSD). While in comparison to WT ST mice RER was increased in Ambra1 ST mice (Ambra1 ST vs WT ST, p< .001 Tukey HSD), Ambra1 mice that underwent CR showed a decrease of RER, indicative of a significant shift towards a prevalent fat oxidation (Ambra1 CR vs Ambra1 ST, p< .0001 Tukey HSD; Fig 3B). However, despite the exposure to CR produced a higher fat and proteins oxidation, the decrease of RER was significantly lower in WT than in Ambra1 mice (WT CR vs Ambra1 CR, p< .0001 Tukey HSD; Fig 3B).

Finally, to identify potential metabolic biomarkers of neuropathic pain development and evaluate CR-induced alteration of Ambra1 metabolic signature, we performed blood analysis of AAs and ACCs (Fig 3C). After CCI, we observed lower levels of C10 and C18 and higher levels of C18:1OH and Orn, in WT ST group, as compared to WT BL mice (S1 Table). By contrast, in the same ST condition, Ambra1 mice showed higher ACCs levels such as short-chain ACCs (C2, C6), odd-chain ACCs (C3, C5), 3-hydroxy and dicarboxy ACCs (C4OH/C3DC, C5DC/C6OH, C6DC, C14OH, C16OH, C18OH), and medium and long-chain ACCs (C8, C10:1, C18:2), as well as higher levels of branched-chain AAs (BCAAs) (Val, Leu/Ile/Pro-OH) and Tyr (Fig 3C; S1 Table), as compared to Ambra1 BL mice. Lower levels of C0 and higher levels of medium-chain ACCs (C6, C8, C10:1, C10) and histidine (His) were detected in WT that underwent CR, when compared to WT in ST regimen (Fig 3C; S4 Table). CR reverted some changes in ACCs, AAs and BCAAs metabolism found in Ambra1 mice. Lower levels of long-chain ACCs (C14, C16, C18:2, C18), 3-hydroxy and dicarboxy-ACCs (C4OH/C3DC, C6DC, C16OH, C18OH), BCAAs (Val, Leu/Ile/Pro-OH), and Tyr (Fig 3C; S4 Table) were found in Ambra1 mice that underwent CR, as compared to Ambra1 ST mice.

### Caloric restriction counteracts neuropathy by enhancing autophagy in Schwann cells: effects on remyelination

As previously demonstrated [13], SCs autophagy played a fundamental role in prevention of pain chronification, and enhancement of autophagic flux had a beneficial effect in the recovery from peripheral neuropathy.

**Fig 4:**
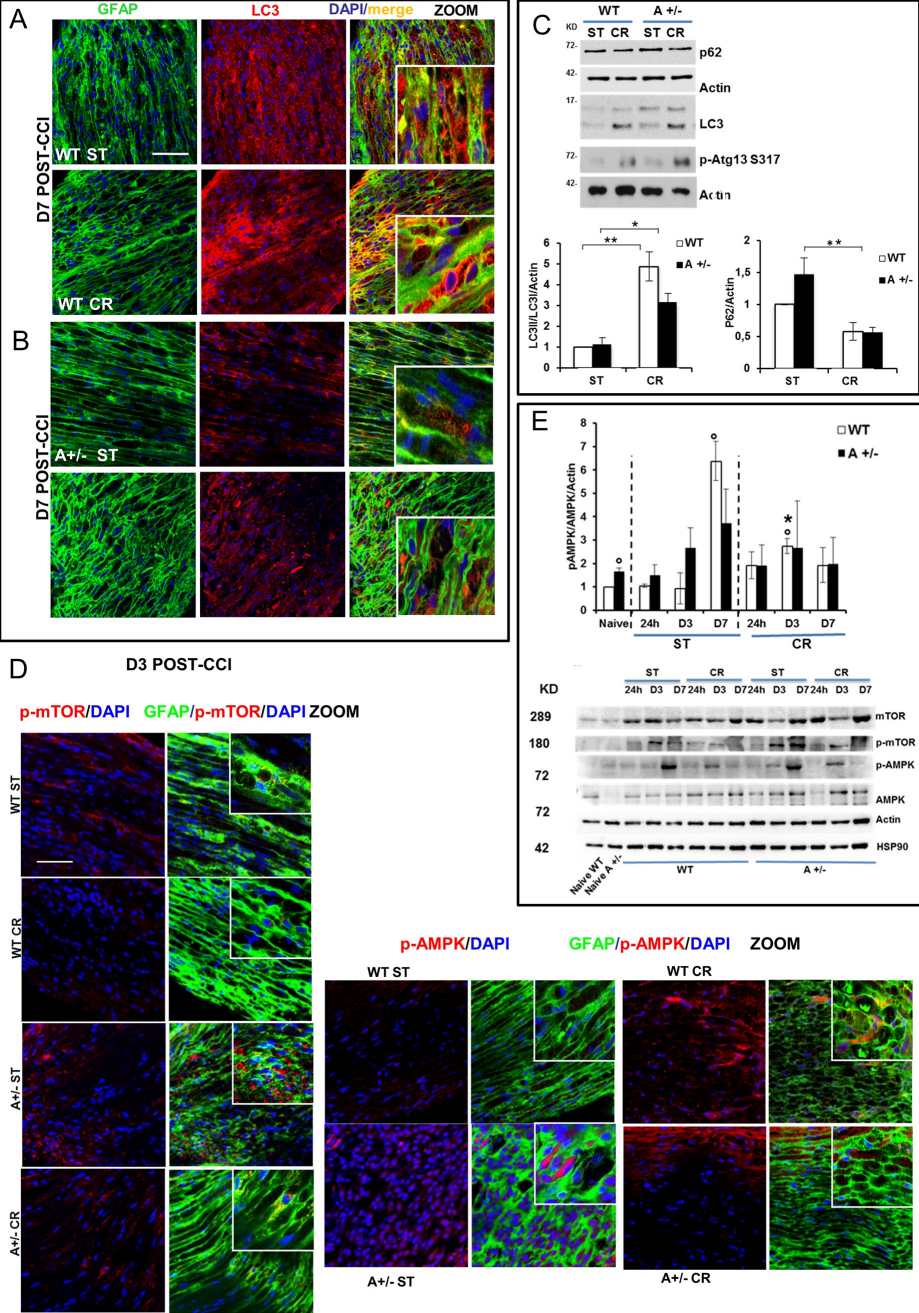
Autophagy evaluation in Schwann cells. Representative pictures of LC3 (red) expression in SCs (GFAP–green) 7 days after CCI, in WT (**A**) or in Ambra1 (**B**) mice both in ST and CR condition; scale bar = 60 micron. (**C**) Western blot (WB) analysis for autophagic proteins (p-Atg13, p62 and LC3II) and quantification in the graph of LC3-II protein in ipsilateral ST vs ipsilateral CR nerves as ratio between LC3-II/actin proteins (P < 0.05). (**D**) Sample images of SCs (GFAP–green) expressing p-mTOR (red) or p-AMPK (red) 3 days after ligature in WT mice in ST diet or CR regimen. (**E**) Proteins, extracted from naïve or ipsilateral sciatic nerves from WT and A+/- mice at 24h, 3 and 7 days from ligature in ST or RC condition, were analysed by WB using anti-pAMPK (T172), anti-AMPK, anti-p-mTOR (S2448), anti-mTOR. Both Actin and Hsp90 were used as loading controls. Densitometric analysis of p-AMPK over Actin is also shown. *P <0.05, **P<0.01, ***<0.0001 vs naïve).

To validate CR-induced autophagy, we analyze different autophagy markers by both immunofluorescence (IF) and Western Blotting (WB) analyses of sciatic nerves. All IF images related to CTRL animals are reported in supporting information. We found a marked LC3 immunostaining in sciatic nerves of animals subjected to CR as compared to ST diet, more evident in WT than in Ambra1 mice (Fig 4A and 4B and S4 Fig). To confirm the IF analysis, we observed a significant increase in LC3-I to LC3-II conversion (H_3_ = 9,46 p .023; WT CR vs WT ST t_4_ = 5,486 p .0054; A+/- CR vs A+/- ST t_4_ = 4,158 p .014; Fig 4C) together with an increase in p-ATG13 protein and a decrease in p62 protein levels (H_3_ = 9,39 p .024; WT CR vs WT ST t_4_ = 2,86 p .045; A+/- CR vs A+/- ST t_4_ = 3,29 p .03) after CR regimen (Fig 4C), suggesting autophagy induction in both genotypes. As metabolic sensor, AMPK is strongly influenced by nutrition status. Interestingly, in Ambra1 naïve animals, p-AMPK is highly expressed as compared to WT naïve (t_4_ = 5,02 p .0074), supporting the idea that myelin aggregates already exist in basal conditions. The status of phosphorylation of mTOR (p-mTOR) and AMPK (p-AMPK) showed that AMPK signaling mediates CR-induced autophagy in sciatic nerves (Fig 4D and 4E). While an increase of p-AMPK was observed at D7 in WT ST animals subjected to CCI (t4 = -6,531 p .0028), no significant differences were detected in Ambra1 mice which, even showing a trend of enhancement, were at the same levels of naïve animals. CR produced an early occurrence of p-AMPK at D3 in WT animals (t4 = 5,19 p .006; Fig 4E) but not in Ambra1 mice, in which the sustained level of p-AMPK as observed in naïve animals, corroborated the existence of elevated basal level of AMPK phosphorylation.

**Fig 5:**
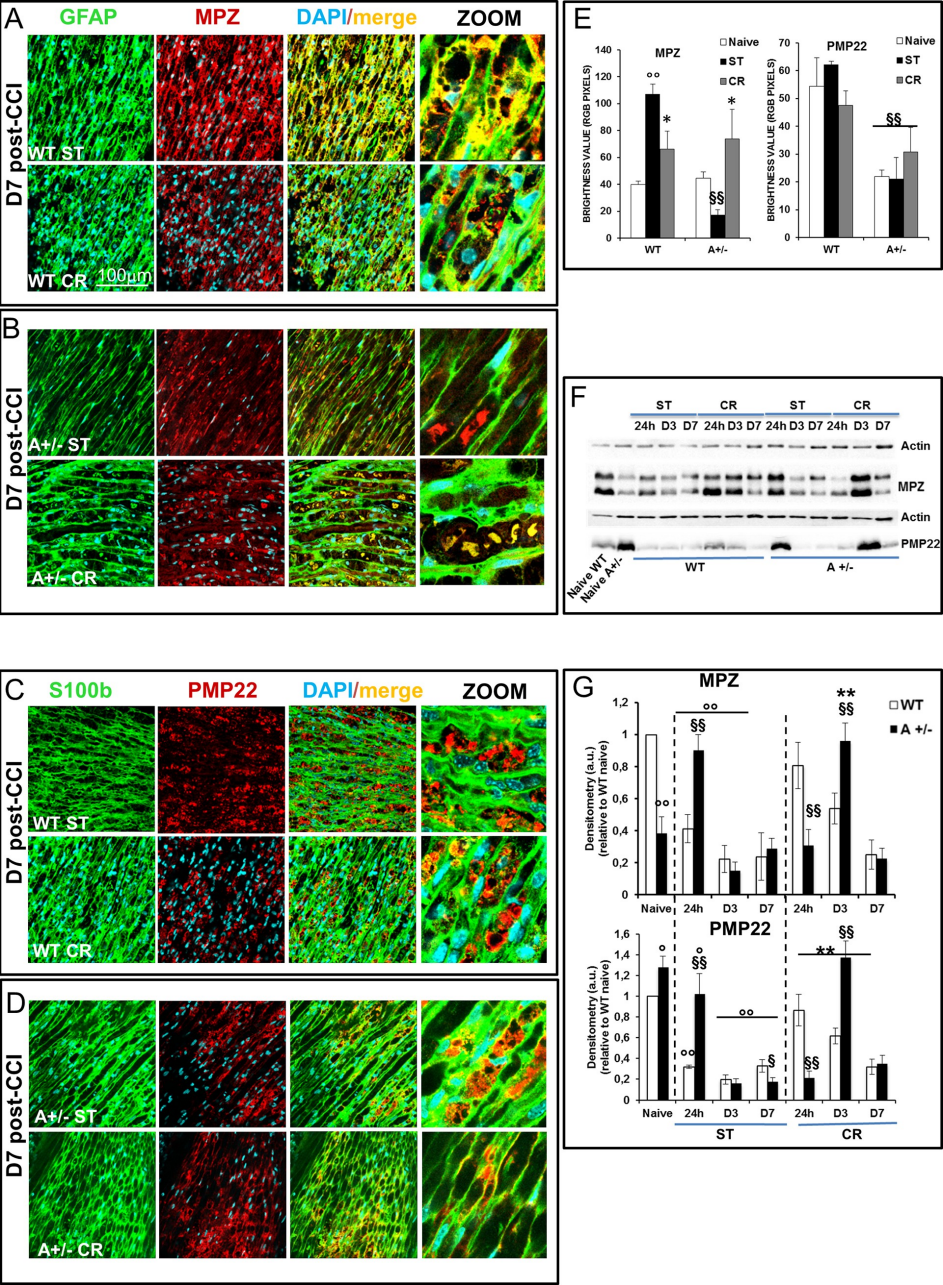
Effects of CR-induced Schwann cells autophagy on remyelination. Expression of myelin protein 0 (MPZ–red) in SCs (GFAP–green), in sciatic nerves derived from WT (**A**) and Ambra1 mice (**B**) in ST or CR condition 7 days after CCI; co-localization (merge–yellow) indicates myelinated fibers. (**C**) Fibers of sciatic nerves in WT and (**D**) in Ambra1 mice in ST and CR regimen 7 days after CCI, double marked with S100b (SCs—green) and the peripheral myelin protein 22 (PMP22 –red). (**E**) Evaluation of MPZ and PMP22 expression. MPZ is overexpressed 7 days after CCI in WT ST vs Naïve (°°P<0,001) and decreased in CR vs ST (*P<0,05); in Ambra1 mice MPZ is down-expressed (^**§§**^P<0,001 vs WT ST) and ameliorated by CR (*P<0,05 CR vs A+/- ST). PMP22 is down-expressed in Ambra1 mice in all conditions considered (^**§§**^P<0,001 A+/- vs WT). (**F**) Proteins extracted from naïve or ipsilateral sciatic nerves from WT and A+/- mice at 24h, 3 and 7 days after CCI in ST or CR condition were analysed by WB using anti-PMP22 and anti-MPZ. Actin is used as loading control. (**G**) Densitometric analyses of MPZ and PMPM22 over Actin are shown. Data are expressed as the mean value±SEM (n = 3) and were analysed by two-way ANOVA followed by Bonferroni’s multiple comparison *post hoc* test.°P<0.05,°°P<0.001 vs naive; §P<0.05, §§P<0.001 vs WT; *P<0.05, **P<0.001 vs ST).

**Fig 6:**
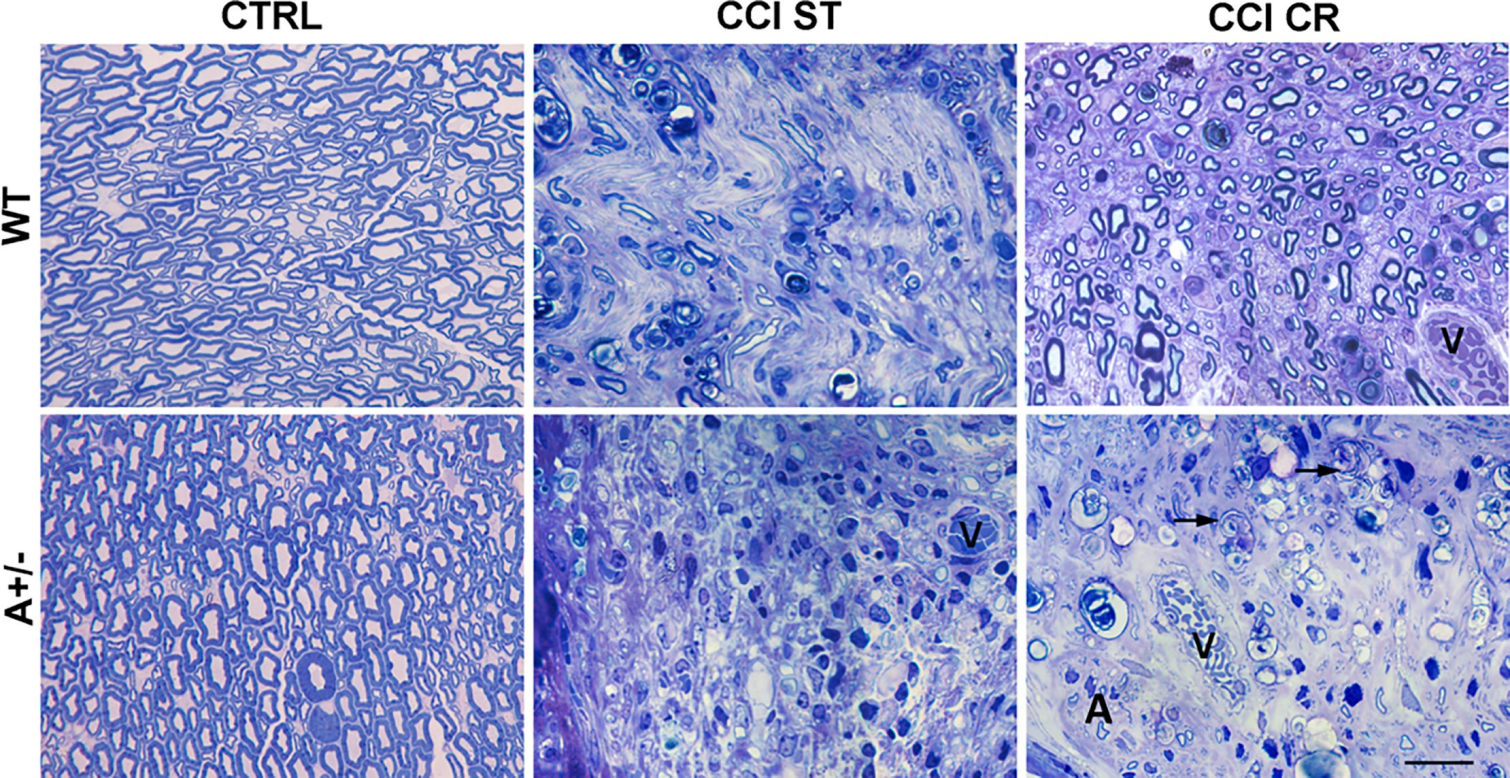
Effects of nerve injury and CR on myelin. Morphological changes in the distal part of sciatic nerves in semithin cross sections from WT and AMBRA1 mice, CTRL or 7 days after CCI. Mice were analysed following ST or CR conditions (0.5 μm thick—magnification 100x; black arrows = large phagocytic cells; v = blood vessel; A = cell in autophagic state; scale bar = 20μm).

To assess myelin changes and its distribution before (naïve) and after injury (D7) we monitored co-staining of glial fibrillary acidic protein (GFAP) with myelin protein zero (MPZ), and the peripheral myelin protein of 22 kDa (PMP22), which plays a role in the maintenance of myelin integrity (control images are reported in S5 Fig). CCI exacerbated myelin degeneration in sciatic nerves of WT and Ambra1 mice in ST conditions (Fig 5A–5D). In WT naïve animals, MPZ and PMP22 were regularly distributed along and inside the fiber (S5 Fig), while in CCI condition both proteins were found aggregated and accumulated inside sciatic nerves (as shown in Fig 5A and 5C and S5 Fig) and, as detected by enhancement of fluorescence, with respect to control nerves (Fig 5E), mirroring the progression of Wallerian degeneration 7 days after lesion. To the other hand, Ambra1 ST mice revealed a reduction in MPZ and PMP22 expression in comparison to WT ST (MPZ: H_5_ = 19,73 p .0014; PMP22: H_5_ = 15,96 p .0069; Fig 5F), which is ascribable to the degenerative status (Fig 5A–5D), as later confirmed by WB proteins analysis (Fig 5E WT ST vs A+/- ST, D7) and morphological analysis (Fig 6). CR stimulated myelinogenesis both in WT and Ambra1 mice, as demonstrated by the reduction of myelin aggregates and from changes in MPZ expression (WT CR vs WT ST t_11_ = 2,76 p .018; A+/- CR vs A+/- ST t_6_ = 2,51 p .045), while no differences in PMP22 expression (always reduced in Ambra1 mice) were visible (Fig 5A–5D and 5E). Increased phagocytosis might explain the reduction of myelin aggregates in Ambra1 mice (Fig 5B; A+/- CR) as also shown by the histological samples (Fig 6). Immunofluorescent data are corroborated by WB analysis of myelin proteins (Fig 5F and 5G), which demonstrated a time-dependent variation of MPZ (H_13_ = 38,65 p .0002) and PMP22 (H_13_ = 38,83 p .0002) after CCI. Of note, Ambra1 naïve mice showed a significant reduction of MPZ (t_4_ = -10,371 p .0005) and an overexpression of PMP22 (t_4_ = 4,71 p .0092) in comparison to WT naïve (Fig 5H), indicating a basal impairment in myelin proteins level. A marked time-dependent decrease of MPZ (t_4_ = 11,33 p .0003) and PMP22 (t_4_ = 63,07 p < .0001) were observed after 24h after CCI in WT ST animals, while in Ambra1 ST mice this decrease was found only from D3 (t4 = -2,972 p .041); a delay that appear imputable to the slower autophagic flux. CR drastically changed the profile of expression of myelin proteins. In WT CR, both MPZ and PMP22 at 24h, resulted enhanced in comparison to WT ST (MPZ: t_4_ = 4,36 p < .012; PMP22: t_4_ = 6,15 p .0035), reaching the same level of WT ST at D7. On the other hand, myelin proteins of Ambra1 in CR regimen were downregulated at 24h in comparison to Ambra1 ST (PMP22: t4 = -6,83 p .0024), then strongly upregulated at D3 (MPZ: t_4_ = 2,67 p .05; PMP22: t4 = 12,66 p .0002) and decreased at D7 as in WT.

These data were supported by the morphological analysis of nerve after CCI (Fig 6) versus CTRL mice. As expected, WT mice in ST condition showed an altered morphology of nerve structure (WT CCI ST), that was almost completely regenerated in WT mice after CR condition (WT CCI CR), resembling WT CTRL mice. Although the cross section of sciatic nerve of CTRL WT and Ambra1 mice did not present any apparent difference (WT CTRL vs A+/- CTRL), with normal myelinated fibers, the sciatic nerves of Ambra1 +/- mice after CCI appeared markedly degenerated and characterized by the massive presence of phagocytes (A+/- CCI ST). Although CR in Ambra1 mice did not induce evident sign of regeneration, except for rare myelinated profiles (A+/- CCI CR), we observed several phagocytes and some SCs in autophagic state, suggesting that an active phase of WD was still occurring.

To further support remyelination of sciatic nerve, we investigated different regenerative markers as shown in S5 Fig.

### Caloric restriction activates anti-inflammatory pathways: Cytokines and chemokines modulation

A panel of 40 pro- and anti-inflammatory mediators was used to verify the effects of CR on cytokines and chemokines in lysates of sciatic nerves and serum at D7 post CCI (Table 1A and 1B; S5 Table). After CCI (S6A and S6B Table—WT naïve vs CCI) the following agents were found up-regulated in nerve lysates: CCL11, GCSF, IL-1β, IL2, IL6, TIMP-1 and TNFr-I. In the serum, the following factors were up-regulated: CD30LG, Eotaxin-1, Eotaxin-2 and GCSF (chemokines), while others were down-regulated: IL1β, IL6, IL12p40/p70 and TNFα and its soluble receptors (Type I and II), KC and MIP1-alpha. After exposition to CR regimen significant changes in pro- and anti-inflammatory cytokines/chemokines expression were reported (Table 1A and 1B). As far as the nerve lysates are concerned, we found a significant up-regulation of the following factors: BLC, CCL24, GM-CSF, IFN-γ, TECK, TNFr-I and TNFr-II; whereas GCSF was drastically decreased. In serum BLC, CXCL1, IL12 and MIG were up-regulated, while IL1β, Eotaxin-2, Fas ligand, IL13, KC and Leptin down-regulated.

**Table 1:** Cytokines modulation.

**a) Nerve Lysate WT CCI D7 ST vs WT CCI D7 CR**		
**Mediator**	**Function**	**Fold change**
BLC	potent chemoattractant for B lymphocytes	1,98
Eotaxin-2 (CCL24)	chemotactic for basophils, Th2 lymphocytes, and tryptase-chymase mast cells	2,48
Granulocyte-macrophage colony-stimulating-factor (GM-CSF)	stimulates growth of progenitors of mono, neutro-, eosino- and basophils; activates macrophages	1,64
IFN-γ	pro-inflammatory; activate macrophages	1,68
Thymus expressed chemokine (TECK)	induces the migration of monocytes and other cell types such as NK cells and dendritic cells	1,69
Soluble TNF (sTNF) receptor I	endogenous inhibitors of TNF	2,07
Soluble TNF (sTNF) receptor II	endogenous inhibitors of TNF	10,47
Granulocyte colony-stimulating factor (GCSF)	stimulates growth of neutro progenitors	-3,37
**b) Serum WT CCI D7 ST vs WT CCI D7 CR**		
**Mediator**	**Function**	**Fold change**
BLC	potent chemoattractant for B lymphocytes	1,67
Fractalkine (CX3CL1)	potent chemoattractant activity for T cells and monocytes; released from apoptotic lymphocytes to stimulate macrophage chemotaxis	1,73
IL-12 p40/p70	inflammatory cytokine inducer	1,60
Monokine induced by gamma interferon (MIG) (CXCL9)	induces the migration of neutrophils; acts as neurotrophic factor promoting neurite outgrowth	5,30
IL-1 beta	induces IL-1,6,8,TNF, GM-CSF by macrophages; proinflammatory	-1,58
Eotaxin-2 (CCL24)	chemotactic for basophils, Th2 lymphocytes, and tryptase-chymase mast cells	-2,64
Fas Ligand	belongs to the tumor necrosis factor (TNF) family and induces apoptosis	-2,46
Il-13	induces and mediates allergic inflammation	-2,29
Keratinocyte-derived chemokine (KC)/chemokine (C-X-C motif) ligand 1	recruits and activates leukocytes	-1,54
Leptin	regulates energy intake and energy expenditure	-19,51

The tables show the significant decrease/increase levels of cytokines analyzed both in nerves tissue lysates samples and in blood. Data are shown as FOLD CHANGE (CCI (ST)/NAIVE or CCI CR/CCI ST). Any ≥ 1. 5-fold increase or ≤ 0. 65-fold decrease in signal intensity for a single analyte between samples may be considered a measurable and significant difference in expression.

## Discussion

In our previous study [13], we showed that SCs autophagy is a primary mechanism involved in the degradation of myelin proteins after nerve injury, and that defective autophagy produced a severe impairment in the recovery from neuropathy. Since autophagy can be mimicked by nutrient deprivation, we used CR as autophagy inducer against NeP chronification. However, CR represents also a metabolic intervention and to understand how metabolic changes affect the response to nerve injury and pain we analyzed different metabolic parameters both in WT (CD1 mice) and in Ambra1 mice. We disclosed the existence of a prediabetic condition in autophagy-defective Ambra1 mice and that nerve damage induced *per se* whole-body metabolic changes.

Prediabetes, or borderline diabetes, is associated to a higher risk of developing diabetes. Prediabetes occurs as an early metabolic dysregulation of glucose metabolism including impaired fasting glucose and/or glucose intolerance with, but also without, insulin resistance over the course of diabetic pathogenesis [32]. Investigating in fasted Ambra1 mice the metabolic parameters correlated to clinical diagnosis of prediabetes, we found slight hyperglycemia, intolerance to glucose (GTT), insulin resistance (high insulin basal levels and ITT impairment) and hyperglucagonemia. It is worth noting that glucose homeostasis is tight regulated by the reciprocal control exerted by insulin and glucagon circulating levels, and that hyperglucagonemia is recognized to account for hyperglycemia and diabetes development [33]. The prediabetic phenotype is also supported by the identification of blood ACCs and AAs as indirect markers of abnormal fatty acid and protein catabolism. Ambra1 mice showed higher levels of long-chain 3-hydroxy ACCs, Orn, and Asn, and lower levels of C3 odd-chain AAs, which is a metabolic profile predictive for the future risk of developing type 2 diabetes (T2DM), as recently found in a population-based prospective study [34]. Notably, the best predictive ability for the increased incidence of T2DM was associated with the increase of long-chain ACCs thus corroborating the idea of a possible dysfunction of FAO and impaired tricarboxylic acid cycle as early dysfunctions in prodromal stage of diabetes. An association between alteration of ACCs levels and dysfunction in glucose metabolism has been found in prediabetic subjects [35], and the higher concentration of long-chain ACCs as marker of possible incomplete FAO has been repeatedly confirmed in prediabetic and newly diagnosed T2DM patients [36, 37].

Together with the prediabetic phenotype, we found that energy metabolism is altered in Ambra1 mice in which energy expenditure is enhanced in resting condition (REE) as well as the level of oxidation of dietary fat are decreased. As for the management of body weight, reduced fat oxidation and increased REE may be viewed as complementary factors. While the decrease of serum triglycerides may contribute to explain the reduction of fat oxidation, it appears that lower oxidation of dietary fat and higher REE compensate each other so that only a slight tendency to body weight increase was observed. As reported in numerous studies [38] increased REE accounts for an obese phenotype and/or for a predisposition to obesity, therefore common also in subject with prediabetes.

Hence, we hypothesized that prediabetes in Ambra1 mice may be a possible causal factor in the exacerbation of CCI-induced neuropathy and chronic pain [13] and that by increasing autophagy we could totally or partially relieve the neuropathic condition.

During the analysis of metabolic changes induced by CR, we first observed that peripheral nerve injury acts as a metabolic stressor. Changes in glycemia, insulin, glucagon, triglycerides, EE and in ACCs were found following CCI also in normometabolic mice. Nerve damage activates several metabolic processes as previously demonstrated in other neurological conditions such as clinical and experimental traumatic spinal injuries [39]. We posit that metabolic changes associated with nerve tissue injury facilitate chronic pain and inflammatory processes. In this view, CR can promote faster recovery by preserving homeostasis and reducing CCI-induced metabolic changes.

In fact, the exposure to CR not only drastically improved response to pain but also changed the prediabetic profile of autophagy-defective mice and CCI-induced metabolic alterations. CR induced an early recovery from allodynia in WT mice (i.e., the ipsilateral allodynic threshold reached the contralateral value at D45) and a decrease of allodynic response in Ambra1 mice that approximate at D45 the spontaneous recovery observed in WT animals.

Nerve injury induced an increase of EE in Ambra1 mice in ST conditions and in both WT and Ambra1 mice that underwent CR. Moreover, while WT CR mice showed decreased EE in resting condition (REE), the metabolic rate was always increased in autophagy-defective mice, irrespective of dietary regimen and motor activity. Oxidation of dietary fat, already reduced in Ambra1 mice before the CCI, was further reduced after nerve injury. By contrast, CR induced an increase of lipid substrate oxidation in both genotypes, which was less marked in Ambra1 animals. In Ambra1 mice, neuropathy induced an increase of different ACCs such as short-chain, odd-chain, 3-hydroxy and dicarboxy and medium and long-chain ACCs, as well as an increase of aromatic AA Tyr, BCAAs and direct products of BCAA catabolism. Previous studies have revealed increased levels of ACCs in T2DM and insulin resistance, supporting the idea of an incomplete FAO as a metabolic footprint of diabetes [36, 37]. Indeed, elevation in aromatic AAs, BCAAs and related metabolites have been described as a “metabolic signature” for insulin resistance, glucose intolerance and obesity [36, 40].

CR improved the metabolic dysregulation found in neuropathic Ambra1 mice. Selective long-chain acylcarnitines, some 3-hydroxy and dicarboxy-acylcarnitines, and BCAAs and Tyr were found reduced in Ambra1 mice subjected to CR. There is evidence for a pathogenetic link between increased BCAAs plasma levels [40, 41], persistent activation of mTOR, ribosomal protein S6 kinase 1 and higher risk of developing insulin resistance and T2DM. For instance, it is recognized that leucine can stimulate the release of glucagon and insulin secretion [42], and that leucine-enriched diet mediates the overstimulation of mTOR signaling and, in turn, S6K1-mediated insulin resistance [43–45].

Since CR is a well-known autophagy inducer, we decided to evaluate its effect on neuropathy, stimulation of myelin debris clearance and remyelination processes. CR strongly activates early autophagy in WT and Ambra1 mice, as supported by the marked LC3 immunostaining in CCI-sciatic nerves, as well as by the increase in LC3-I to LC3-II conversion. However, while in WT animals this increase occurred via mTOR inhibition and p-AMPK, the slight improvement in myelin clearance observed in Ambra1 mice appeared attributable to CR-induced macrophages activity (A+/- CR, Fig 5B and 5E). CR-induced autophagy has beneficial effects on the trophic function of SCs, and we showed that the increase of proteins associated with axon re-growth and SCs proliferation is part of the recovery process, as for the early autophagy of myelin debris to repair lesioned axons and production of new myelin, confirming myelinophagy as a major mechanism for SCs-mediated myelin clearance after nerve lesion [4] and demonstrating the ability of SCs to respond to metabolic signals.

Since CR improved both neuropathy-associated inflammatory micro-environment and metabolic profile, we analyzed cytokines and chemokines in response to CR regimen. As chronic pain biomarker [46, 47], TIMP-1 resulted up-regulated by CCI and normalized by the exposure to CR. There is also evidence for an anti-inflammatory role for AMPK [48, 49] and several molecules involved in the stimulation and regulation of IFN-γ such as IL-12, MIG, TNF-α receptors (I-II) were found upregulated in serum and lysates from animals subjected to CR. IFN-γ participates in mTORC1 downregulation [50], and its increase corroborates the activation of autophagy machinery and macrophage activity. In the CNS, TNF-α may have either neurotoxic or neuroprotective potential, and this dual role is believed associated with the activation of pro- or anti-apoptotic transduction pathways by the different TNF-α receptor subtypes [51, 52]. We believe that CR may reduce TNF-α pro-inflammatory activity by facilitating TNF-α RII activity over that of TNF-α RI via IFN-γ-mediated regulation [53].

## Conclusions

Taken together, our results show that autophagy deficiency can “host” prediabetes and that the dysmetabolic profile of Ambra1 mice fits well with the early stage of clinical diabetes. We demonstrated that these conditions of borderline diabetes and defective autophagy are the mechanisms responsible for the exacerbation of allodynia previously showed in Ambra1 mice. Also, these data provide evidence that CR-induced stimulation of autophagy machinery, can exert an effective painkiller function against neuropathy. Moreover, AMPK-mediated autophagy, myelinogenesis and selective anti-inflammatory mechanisms play a causative role in this process. Finally, the ability of CR to rebalance the alterations of ACCs as possible markers of incomplete long-chain FAO in Ambra1 mice reveals the complex relationship among autophagy, dietary factors, dysfunction of lipid and glucose metabolism and diabetes.

## Supporting information

S1 Fig Sex-related differences in neuropathic pain development in WT and Ambra1 mice. Graph shows that all animals developed neuropathic pain (IPSI vs CONTRA°°° = p<0.0001) and no differences were found in allodynic response between male (M) and female (F) Ambra1 (A+/-) mice. A+/- F were significantly different from WT F (* = p<0.05 and *** = p<0.0001). Our previous data (Vacca et al. 2014, 2016) already demonstrated the different response to nerve damage between WT male and female, as here confirmed.
(TIF)

S2 Fig PLS-DA score plot based on whole blood amino acid (AA) and acylcarnitine (ACC) concentrations found at baseline in wild type versus Ambra1 mice (WT BL vs A+/- BL). (TIF)

S3 Fig Data on the volume of O2 consumption (VO2). An increase of VO2 (ml/h) was observed in WT CR, Ambra1 ST and Ambra1 CR but not in WT ST animals. (°P<0.05°°P<0.001 vs WT; *P<0.05 **P<0,001 vs ST diet; §P<0.05 §§P<0,001 vs A+/-).
(TIF)

S4 Fig Effects of CR on Schwann cells autophagy. Autophagy is evaluated by means of LC3 staining (RED). LC3 is normally expressed in Schwann cell (GFAP–green) in basal condition (CTRL). 7 days after CCI (CCI ST) or after the period of CR, dots of LC3 are evident, and indicate that cells undergoing autophagic. The evaluation of LC3 expression (brightness values) demonstrates the effect of treatment (H5 = 17,871 p 0.0031), an increase of autophagy after CCI and CR in WT mice (°°p<0.001 and°°°p<0.0001 vs CTRL), an impairment of Ambra1 mice (A+/-) in Schwann cell autophagy with respect to WT (§p<0.05 and §§ p<0.001 vs WT) and the improvement after CR (°p<0.05 vs CTRL).
(TIF)

S5 Fig CCI-induced myelin degeneration and CR-increased remyelination. Sample images (magnification 63X) of myelin markers (A) GFAP/MPZ and (B) S100b/PMP22 merge and relative zoom (2X), allowing to appreciate morphological and structural changes that occurs after CCI and in response to CR.
(TIF)

S6 Fig Effect of CR on sciatic nerve regenerative and structural proteins. Regenerative processes in SCs were evaluated via the staining of different markers: cell division cycle protein 2 (CDC2), a mitotic cyclin; neurofilament 200 (NF200), a cytoskeletal protein of myelinated axons and growth associated protein 43 (GAP43), an axonal membrane protein. Representative confocal IF images of SCs (GFAP–green) in proliferative state (CDC2 –red) in WT (A) and Ambra1 (C) mice sciatic nerves 7 days after CCI. After 7 days from CCI, CDC2, GAP43 and NF200 proteins are highly expressed in damaged nerves in comparison to CTRL animals (confocal images not shown) (B) Bar graph shows significant enhancement of CDC2 expression after CCI in WT ST vs control (CTRL) animals (°P<0,05) and in WT ST vs CR mice (*P<0,05). In Ambra1 mice, CR regimen induced a significant enhancement vs ST and CTRL animals (P<0,05). Sample pictures of CCI sciatic nerves double marked for intermediate neurofilaments (NF200 –green) and axonal growth protein (GAP43 –red) in WT (D) and Ambra1 mice (F). (E) Graph shows a significant expression of GAP43 after CCI (CTRL vs WT ST;°P<0,05) strongly enhanced by CR (**P<0,0001). In Ambra1 mice, GAP43 expression was increased in ST condition vs CTRL (°P<0,05). (G) NF200 expression was significant enhanced 7 days after ligature (CTRL vs WT ST;°°P<0,0001). Ambra1 mice showed any modifications in NF200 expression in all conditions considered.
(TIF)

S1 Table Experimental groups, conditions and group size. (PDF)

S2 Table Whole blood amino acid and acylcarnitine profiling of wild type (WT, n = 12) and Ambra1 (A+/-, n = 12) at baseline and 7 days after CCI in ST dietary regimen. Data are mean concentrations expressed in μmol L-1 and p-values statistically significant (95% confidence level) from two-factor mixed design ANOVA and post-hoc multiple comparisons. SD: standard deviation. NS: not significant.
(PDF)

S3 Table Electrospray ionization mass spectrometry (ESI-MS) acquisition parameters used for the analysis of whole blood amino acids (AAs) and acylcarnitines (ACCs). MS/MS transitions for each analysed AA and ACC and the corresponding internal standard (IS, shown in bold), the optimal cone potential (V), and collision energy (eV) are shown for each analyte. The capillary potential was 3.5 kV.
(PDF)

S4 Table Whole blood amino acid and acylcarnitine profiling of wild type (WT) and Ambra1 (A+/-) 7 days after CCI upon ST (WT ST, n = 12; A+/- ST, n = 7) or CR (WT CR, n = 11; A+/- CR, n = 11) regimen. Data are mean concentrations expressed in μmol L-1 and p-values statistically significant (95% confidence level) from two-factor ANOVA and post-hoc multiple comparisons. SD: standard deviation. NS: not significant.
(PDF)

S5 Table Complete map of RayBioMouse Inflammation Antibody Array G-Series utilized for serum and nerve lysate samples. (TIF)

S6 Table **Tables a) and b) show only the significant decrease/increase levels of cytokines analyzed both in nerves tissue lysates samples and in blood.** Data are shown as FOLD CHANGE (CCI (ST)/NAIVE or CCI CR/CCI ST). Any ≥ 1. 5-fold increase or ≤ 0. 65-fold decrease in signal intensity for a single analyte between samples may be considered a measurable and significant difference in expression.
(PDF)
