# Supplementary figures and images for: Effects of caloric restriction on neuropathic pain, peripheral nerve degeneration and inflammation in normometabolic and autophagy defective prediabetic Ambra1 mice
Source: PLoS One. 2018 Dec 10;13(12):e0208596. doi: 10.1371/journal.pone.0208596 (PMC6287902; doi:10.1371/journal.pone.0208596)

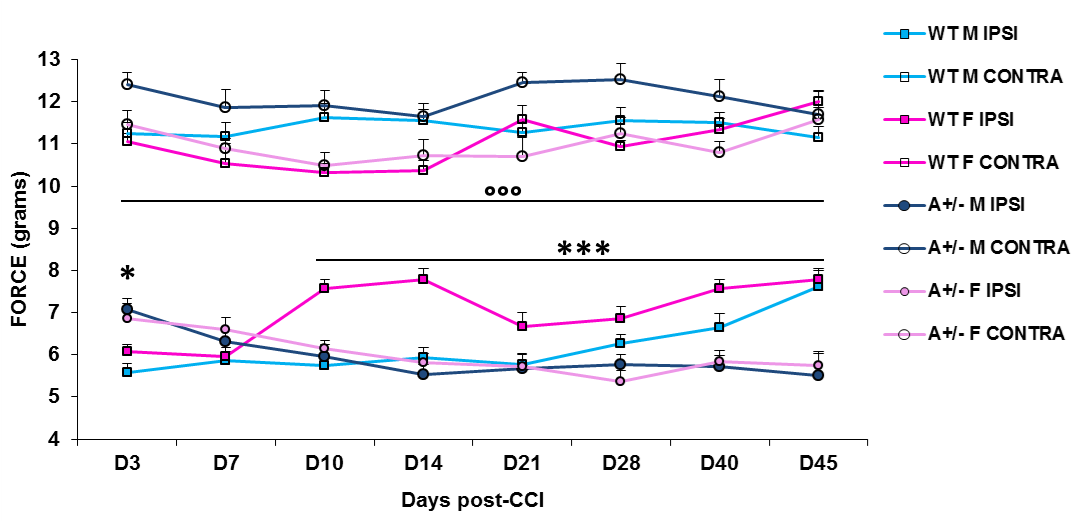

Supplement: S1 Fig — Graph shows that all animals developed neuropathic pain (IPSI vs CONTRA°°° = p<0.0001) and no differences were found in allodynic response between male (M) and female (F) Ambra1 (A+/-) mice. A+/- F were significantly different from WT F (* = p<0.05 and *** = p<0.0001). Our previous data (Vacca et al. 2014, 2016) already demonstrated the different response to nerve damage between WT male and female, as here confirmed. (TIF) [file pone.0208596.s001.tif]

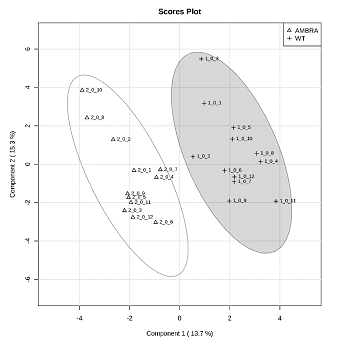

Supplement: S2 Fig — (TIF) [file pone.0208596.s002.tif]

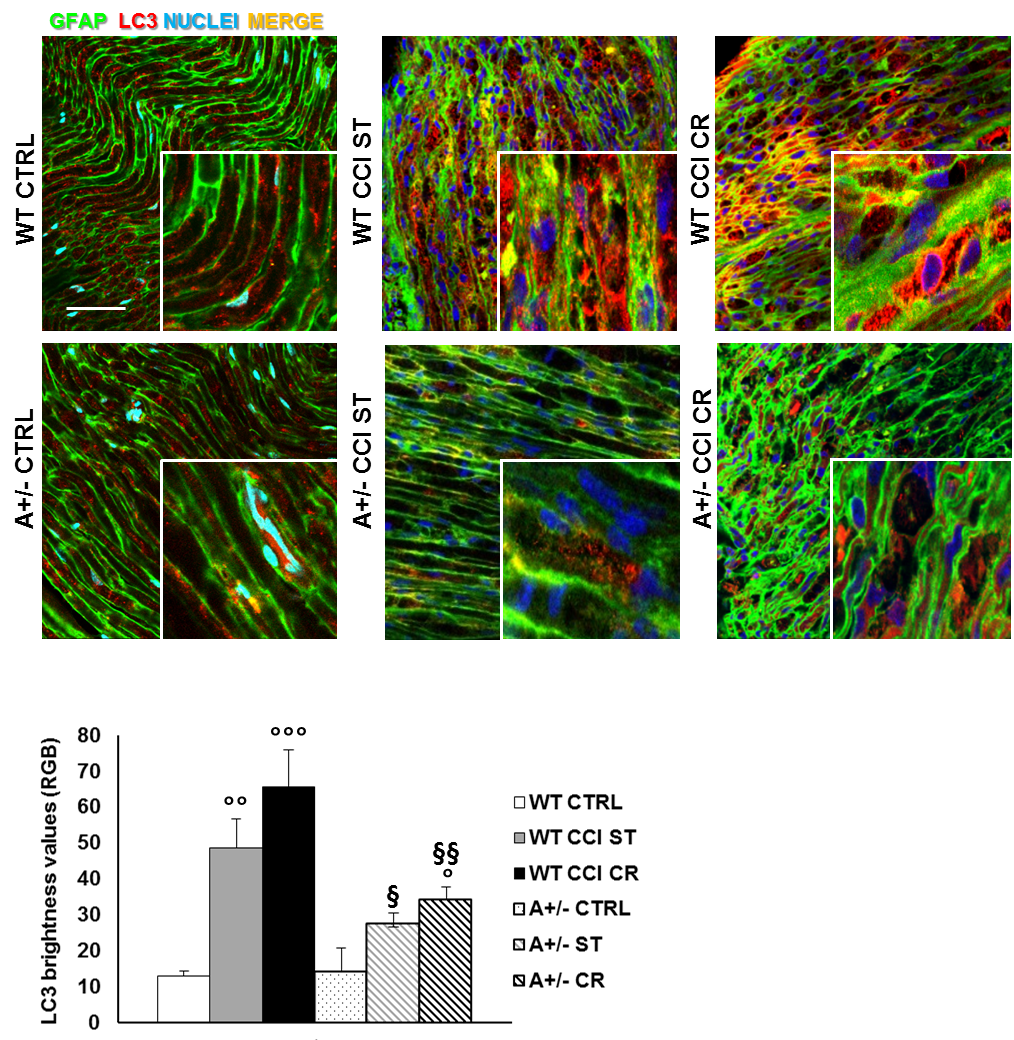

Supplement: S4 Fig — Autophagy is evaluated by means of LC3 staining (RED). LC3 is normally expressed in Schwann cell (GFAP–green) in basal condition (CTRL). 7 days after CCI (CCI ST) or after the period of CR, dots of LC3 are evident, and indicate that cells undergoing autophagic. The evaluation of LC3 expression (brightness values) demonstrates the effect of treatment (H5 = 17,871 p 0.0031), an increase of autophagy after CCI and CR in WT mice (°°p<0.001 and°°°p<0.0001 vs CTRL), an impairment of Ambra1 mice (A+/-) in Schwann cell autophagy with respect to WT (§p<0.05 and §§ p<0.001 vs WT) and the improvement after CR (°p<0.05 vs CTRL). (TIF) [file pone.0208596.s004.tif]

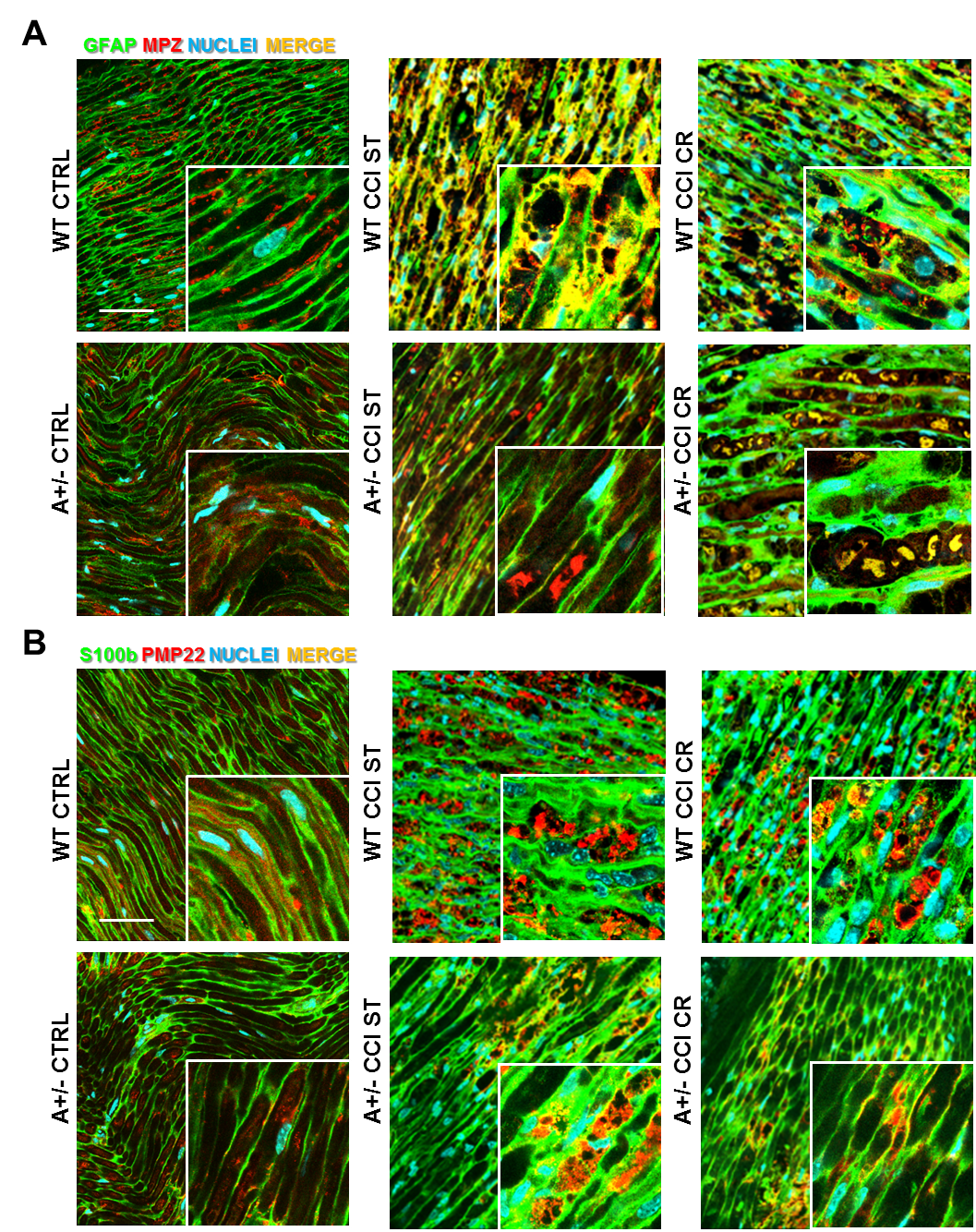

Supplement: S5 Fig — Sample images (magnification 63X) of myelin markers (A) GFAP/MPZ and (B) S100b/PMP22 merge and relative zoom (2X), allowing to appreciate morphological and structural changes that occurs after CCI and in response to CR. (TIF) [file pone.0208596.s005.tif]

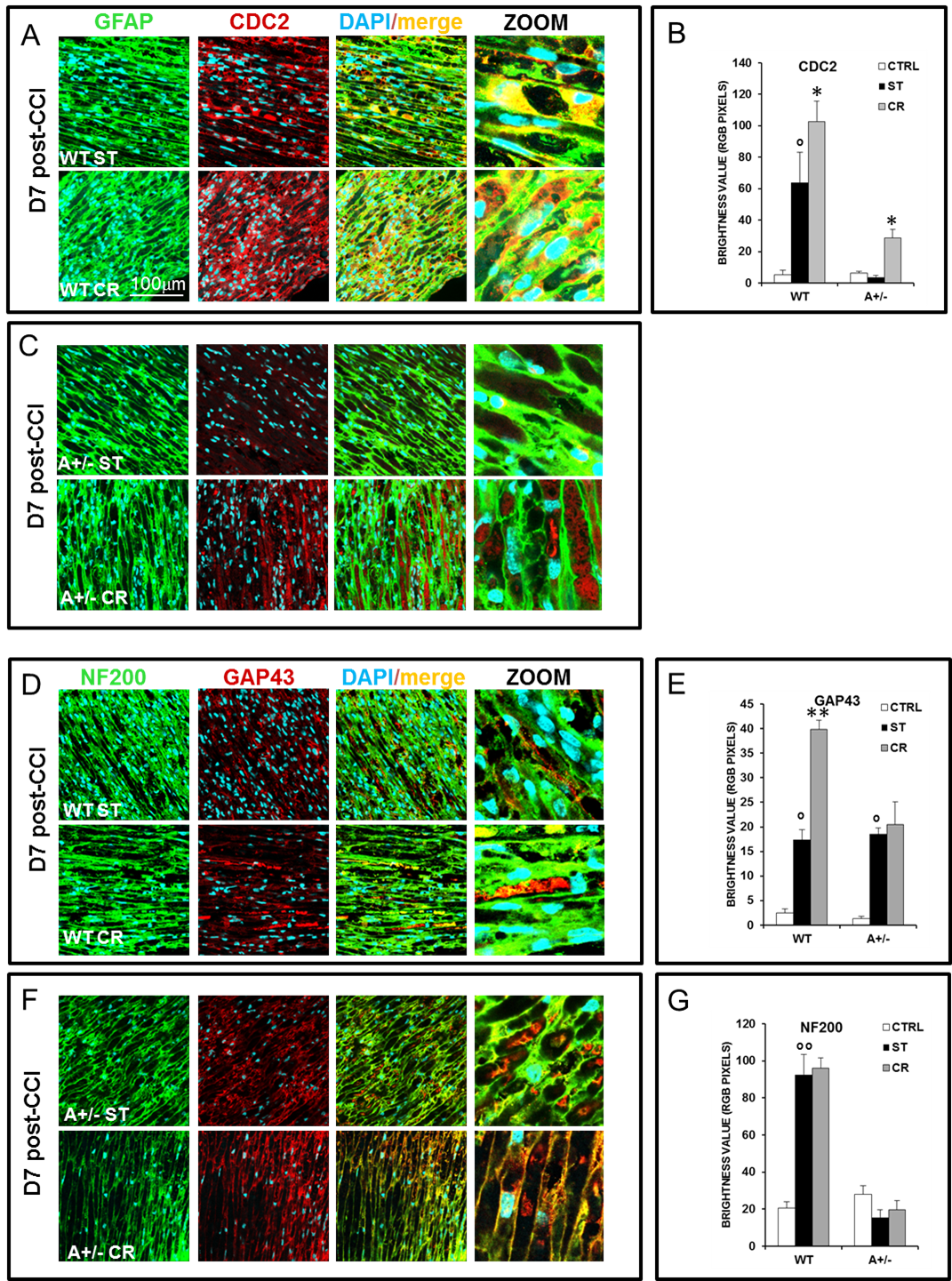

Supplement: S6 Fig — Regenerative processes in SCs were evaluated via the staining of different markers: cell division cycle protein 2 (CDC2), a mitotic cyclin; neurofilament 200 (NF200), a cytoskeletal protein of myelinated axons and growth associated protein 43 (GAP43), an axonal membrane protein. Representative confocal IF images of SCs (GFAP–green) in proliferative state (CDC2 –red) in WT (A) and Ambra1 (C) mice sciatic nerves 7 days after CCI. After 7 days from CCI, CDC2, GAP43 and NF200 proteins are highly expressed in damaged nerves in comparison to CTRL animals (confocal images not shown) (B) Bar graph shows significant enhancement of CDC2 expression after CCI in WT ST vs control (CTRL) animals (°P<0,05) and in WT ST vs CR mice (*P<0,05). In Ambra1 mice, CR regimen induced a significant enhancement vs ST and CTRL animals (P<0,05). Sample pictures of CCI sciatic nerves double marked for intermediate neurofilaments (NF200 –green) and axonal growth protein (GAP43 –red) in WT (D) and Ambra1 mice (F). (E) Graph shows a significant expression of GAP43 after CCI (CTRL vs WT ST;°P<0,05) strongly enhanced by CR (**P<0,0001). In Ambra1 mice, GAP43 expression was increased in ST condition vs CTRL (°P<0,05). (G) NF200 expression was significant enhanced 7 days after ligature (CTRL vs WT ST;°°P<0,0001). Ambra1 mice showed any modifications in NF200 expression in all conditions considered. (TIF) [file pone.0208596.s006.tif]

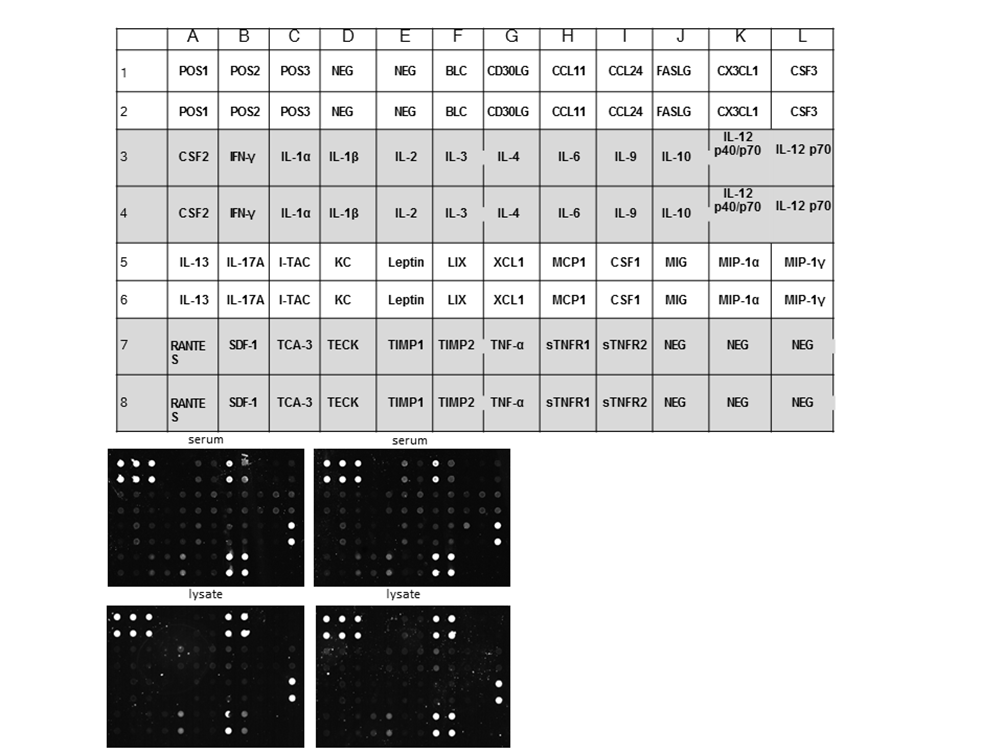

Supplement: S5 Table — (TIF) [file pone.0208596.s011.tif]
