# Supplementary material for: Effects of caloric restriction on neuropathic pain, peripheral nerve degeneration and inflammation in normometabolic and autophagy defective prediabetic Ambra1 mice
Source: PLoS One. 2018 Dec 10;13(12):e0208596. doi: 10.1371/journal.pone.0208596 (PMC6287902; doi:10.1371/journal.pone.0208596)
Supplement: S1 Table — (PDF) [file pone.0208596.s007.pdf]

Experimental Groups	Feeding condition	Measures and size (N per group)	Time-points
**Metabolic phenotype**			
**WT**<br><br>**A+/-**	Naïve: fasted (18h)	Glycemia (N10/11)	-
	Naïve: not fasted	Insulin (N3)	-
	Naïve: not fasted	Glucagon (N3)	-
	Naïve: not fasted	Triglycerides (N14/18)	-
	Naïve: fasted (18h)	Glucose Tolerance Test (N10/11)	T0-30’-60’-120’
	Naïve: fasted (5h)	Insulin Tolerance Test (N8/9)	T0-30’-60’-120’
	Naïve: not fasted	Indirect Calorimetry (N18/22)	From 0 to 48h
	Naïve: not fasted	Metabolomics (N12)	-
**Pre- and Post-CCI**			
**WT ST**<br><br>**WT CR**<br><br>**A+/- ST**<br><br>**A+/- CR**	**Naive**: not fasted;<br><br>**BL**: before CCI not fasted;<br><br>**ST**: after CCI not fasted;<br><br>**CR:** after CCI (40% less of food availability for 7 days).	Aesthesiometer Plantar Test (N11/12)	D3, D7, D10, D14, D21, D28, D40, D45
		Body Weight (N11/12)	BL (pre-CCI), D7
		Glycemia (N5/7)	BL (pre-CCI), 24h, D3, D7
		Triglycerides (N5/7)	BL (pre-CCI), 24h, D3, D7
		Glucagon (N3)	BL (pre-CCI), 24h, D3, D7
		Insulin (N3)	BL (pre-CCI), 24h, D3, D7
		Indirect Calorimetry (N9/11)	BL (pre-CCI), from 0h to 48h after CCI
		Metabolomics (N7/12)	BL (pre-CCI), D7
		IF and WB for LC3, Atg13, p62 (N3)	D7
		IF and WB for AMPK, mTOR (N3)	Naive, D3<br>Naive, 24h, D3, D7
		IF and WB for MPZ, PMP22 (N3)	naive, D3<br>Naive, 24h, D3, D8

Acronyms in table: WT= wild type; A+/-= Ambra1 gt/+ mice; ST= standard diet; CR= caloric restriction; BL= baseline; CCI = Chronic Constriction Injury; IF= immunofluorescence; WB= Western Blot; LC3= Microtubule-associated proteins 1A/1B light chain 3B; Atg13=Autophagy-Related Protein 13; p62= sequestosome 1; AMPK= 5' AMP-activated protein kinase; mTOR= mammalian Target of Rapamycin; MPZ= Myelin protein Zero; PMP22= Peripheral Myelin protein 22.
