# Supplementary material for: Effects of caloric restriction on neuropathic pain, peripheral nerve degeneration and inflammation in normometabolic and autophagy defective prediabetic Ambra1 mice
Source: PLoS One. 2018 Dec 10;13(12):e0208596. doi: 10.1371/journal.pone.0208596 (PMC6287902; doi:10.1371/journal.pone.0208596)
Supplement: S2 Table — Data are mean concentrations expressed in μmol L-1 and p-values statistically significant (95% confidence level) from two-factor mixed design ANOVA and post-hoc multiple comparisons. SD: standard deviation. NS: not significant. (PDF) [file pone.0208596.s008.pdf]

Metabolite	WT BL		WT ST		A+/- BL		A+/- ST		Two-factor mixed design ANOVA, p			Fisher's Least Significant Difference post-hoc test, p		
	Mean	SD	Mean	SD	Mean	SD	Mean	SD	Genotype	CCI	Genotype*CCI	WT BL vs A+/- BL	WT BL vs WT ST	A+/- BL vs A+/- ST
C10	0.068	0.008	0.05	0.01	0.069	0.008	0.079	0.009	<0.001	NS	<0.001	NS	<0.001	NS
C18	0.50	0.05	0.44	0.08	0.49	0.05	0.53	0.09	NS	NS	0.017	NS	0.024	NS
C2	26	5	26	5	25	3	36	12	0.033	0.009	0.013	NS	NS	0.001
C3	0.8	0.1	0.7	0.2	0.6	0.1	0.8	0.1	NS	NS	0.004	0.003	NS	0.003
C5	0.23	0.06	0.25	0.07	0.21	0.04	0.28	0.07	NS	0.037	NS	NS	NS	0.023
C6	0.09	0.02	0.09	0.03	0.09	0.02	0.12	0.02	NS	NS	0.005	NS	NS	0.002
C8	0.09	0.02	0.08	0.02	0.10	0.02	0.12	0.02	<0.001	NS	0.030	NS	NS	0.024
C4OH/C3DC	0.2	0.1	0.24	0.07	0.24	0.05	0.4	0.2	0.011	0.009	NS	NS	NS	0.004
C5DC/C6OH	0.27	0.04	0.32	0.09	0.28	0.08	0.4	0.1	NS	0.004	NS	NS	NS	0.011
C6DC	0.13	0.04	0.14	0.05	0.12	0.05	0.18	0.06	NS	0.016	NS	NS	NS	0.009
C14OH	0.04	0.01	0.05	0.02	0.047	0.008	0.06	0.02	NS	0.018	NS	NS	NS	0.038
C10:1	0.05	0.01	0.05	0.01	0.058	0.009	0.07	0.02	<0.001	0.036	0.021	NS	NS	0.004
C16OH	0.11	0.02	0.15	0.06	0.15	0.03	0.20	0.09	0.011	0.014	NS	0.006	NS	0.043
C18:2	0.3	0.1	0.4	0.1	0.4	0.1	0.6	0.2	<0.001	0.005	NS	NS	NS	0.002
C18:1OH	0.07	0.02	0.11	0.07	0.13	0.03	0.16	0.05	0.001	0.011	NS	0.010	0.043	NS
C18OH	0.043	0.006	0.05	0.02	0.06	0.01	0.08	0.02	<0.001	0.034	NS	0.007	NS	0.034
Short-chain ACCs[a]	26	5	27	5	26	3	36	12	0.033	0.009	0.013	NS	NS	0.001
Odd-chain ACCs[b]	1.0	0.2	0.9	0.2	0.8	0.1	1.07	0.09	NS	0.026	0.002	0.003	NS	<0.001
3-Hydroxy/Di-carboxy ACCs[c]	0.9	0.1	1.1	0.3	1.0	0.1	1.5	0.3	<0.001	<0.001	0.050	NS	0.041	<0.001
Medium&Long-chain ACCs[d]	1.0	0.2	1.0	0.2	1.1	0.1	1.4	0.3	<0.001	0.041	0.008	NS	NS	0.002
Val	107	21	116	21	100	23	134	25	NS	0.004	NS	NS	NS	0.003
Leu/Ile/Pro-OH	173	31	197	49	173	44	239	55	NS	0.002	NS	NS	NS	0.003
Tyr	83	19	79	18	89	35	128	27	0.001	0.035	0.011	NS	NS	0.003
Orn	57	7	75	24	80	25	75	21	NS	NS	NS	0.009	0.034	NS
Asn	2.8	0.5	3	1	4	1	2.9	0.7	NS	NS	0.020	0.041	NS	NS

[a]Sum of C2 and C6 whole blood concentrations (μmol L^{-1}).
[b]Sum of C3 and C5 whole blood concentrations (μmol L^{-1}).
[c]Sum of C4OH/C3DC, C5DC/C6OH, C6DC, C14OH, C16OH, C18:1OH, and C18OH whole blood concentrations (μmol L^{-1}).
[d]Sum of C8, C10:1, C10, C18:2, C18 whole blood concentrations (μmol L^{-1}).
