# Supplementary material for: Effects of caloric restriction on neuropathic pain, peripheral nerve degeneration and inflammation in normometabolic and autophagy defective prediabetic Ambra1 mice
Source: PLoS One. 2018 Dec 10;13(12):e0208596. doi: 10.1371/journal.pone.0208596 (PMC6287902; doi:10.1371/journal.pone.0208596)
Supplement: S3 Table — MS/MS transitions for each analysed AA and ACC and the corresponding internal standard (IS, shown in bold), the optimal cone potential (V), and collision energy (eV) are shown for each analyte. The capillary potential was 3.5 kV. (PDF) [file pone.0208596.s009.pdf]

Abbreviation<br>**IS**	Full name	Transition	Cone potential	Collision energy
Ala<br>**D4Ala**	Alanine	90.2>44.3<br>94.2>48.3	40	6
Arg<br>His<br>**D5Arg**	Arginine<br>Histidine	175.3>70.3<br>156.2>110.2<br>180.3>75.3	45	17
Cit<br>**D2Cit**	Citrulline	176.2>113.2<br>178.2>115.2	45	13
Gly<br>**D2Gly**	Glycine	76.2>30.3<br>78.2>32.3	40	5
Leu/Ile/Pro-OH<br>Asp<br>Glu<br>Asn<br>**D3Leu**	Leucine/Isoleucine/Hydroxyproline<br>Aspartic Acid<br>Glutamic Acid<br>Asparagine	132.2>86.3<br>134.2>88.2<br>148.2>84.2<br>133.2>74.2<br>135.2>89.3	40	8
Met<br>**D3Met**	Methionine	150.2>104.2<br>153.2>107.2	45	9
Orn<br>Lys/Gln<br>**D6Orn**	Ornithine<br>Lysine/Glutamine	133.3>70.3<br>147.2>130.2<br>139.3>76.3	40	12
Phe<br>**D6Phe**	Phenylalanine	166.2>120.2<br>172.2>126.2	45	11
Tyr<br>**D6Tyr**	Tyrosine	182.2>136.2<br>188.2>142.2	45	12
Val<br>Ser<br>Thr<br>**D8Val**	Valine<br>Serine<br>Threonine	118.2>72.3<br>106.1>60.3<br>120.2>74.3<br>126.2>80.3	40	8
C0<br>**D9C0**	Free Carnitine	162.2>103.2<br>171.2>103.2	60	14
C2<br>**D3C2**	Acetylcarnitine	204.2>85.2<br>207.2>85.2	60	14
C3<br>**D3C3**	Propionylcarnitine	218.2>85.2<br>221.2>85.2	60	15
C4<br>C3DC/C4OH<br>**D3C4**	Butyrylcarnitine Malonylcarnitine/3-Hydroxy-butyrylcarnitine	232.3>85.2<br>248.3>85.2<br>235.3>85.2	60	15
C5<br>C5:1<br>C4DC/C5OH<br>**D9C5**	Valerylcarnitine Tiglylcarnitine Methylmalonylcarnitine/3-Hydroxy-valerylcarnitine	246.2>85.2<br>244.2>85.2<br>262.2>85.2<br>255.2>85.2	70	16
C6<br>**D3C6**	Hexanoylcarnitine	260.3>85.2<br>263.3>85.2	65	16

Table S3. Second part

**Abbreviation**<br>**IS**	**Full name**	**Transition**	**Cone potential**	**Collision energy**
C5DC/C6OH<br>C6DC<br>**D6C5DC**	Glutarylcarnitine/3-Hydroxy-hexanoylcarnitine<br>Adipylcarnitine	276.3>85.2<br>290.3>85.2<br>282.3>85.2	70	20
C8:1<br>C8<br>**D3C8**	Octenoylcarnitine<br>Octanoylcarnitine	286.3>85.2<br>288.3>85.2<br>291.3>85.2	75	18
C10<br>C10:2<br>C10:1<br>**D3C10**	Decanoylcarnitine<br>Decadienoylcarnitine<br>Decenoylcarnitine	316.3>85.2<br>313.>85.2<br>314.3>85.2<br>319.3>85.2	75	19
C12<br>C12:1<br>**D3C12**	Dodecenoylcarnitine<br>Dodecanoylcarnitine	344.4>85.2<br>342.4>85.2<br>347.4>85.2	75	22
C14<br>C14:1<br>C14:2<br>C14OH<br>**D3C14**	Tetradecanoylcarnitine (myristoylcarnitine)<br>Tetradecenoylcarnitine<br>Tetradecadienoylcarnitine<br>3-Hydroxy-tetradecanoylcarnitine	372.4>85.2<br>370.4>85.2<br>368.4>85.2<br>388.4>85.2<br>375.4>85.2	75	23
C16:1<br>C16<br>C16:1-OH<br>C16OH<br>**D3C16**	Hexadecenoylcarnitine<br>Hexadecanoylcarnitine (palmitoylcarnitine)<br>3-Hydroxy-hexadecenoylcarnitine<br>3-Hydroxy-hexadecanoylcarnitine	398.4>85.2<br>400.4>85.2<br>414.4>85.2<br>416.4>85.2<br>403.4>85.2	75	25
C18<br>C18:1<br>C18:2<br>C18:1-OH<br>C18OH<br>**D3C18**	Octadecanoylcarnitine (Stearoylcarnitine)<br>Octadecenoylcarnitine (Oleylcarnitine)<br>Octadecadienoylcarnitine (Linoleylcarnitine)<br>3-Hydroxy-octadecenoylcarnitine<br>3-Hydroxy-octadecanoylcarnitine	428.4>85.2<br>426.4>85.2<br>424.4>85.2<br>442.4>85.2<br>444.4>85.2<br>431.4>85.2	80	25
