# Supplementary material for: Effects of caloric restriction on neuropathic pain, peripheral nerve degeneration and inflammation in normometabolic and autophagy defective prediabetic Ambra1 mice
Source: PLoS One. 2018 Dec 10;13(12):e0208596. doi: 10.1371/journal.pone.0208596 (PMC6287902; doi:10.1371/journal.pone.0208596)
Supplement: S4 Table — Data are mean concentrations expressed in μmol L-1 and p-values statistically significant (95% confidence level) from two-factor ANOVA and post-hoc multiple comparisons. SD: standard deviation. NS: not significant. (PDF) [file pone.0208596.s010.pdf]

	WT ST		WT CR		A+/- ST		A+/- CR		Two factors mixed ANOVA, p			Fisher's Least Significant Difference post-hoc test, p	
Metabolite	Mean	SD	Mean	SD	Mean	SD	Mean	SD	Genotype	CCI	Genotype* CCI	WT ST vs WT CR	A+/- ST vs A+/- CR
C0	31	6	25	7	28	3	29	7	NS	NS	0.095	0.032	NS
C4OH/C3DC	00.02	00.01	00.03	00.01	00.04	00.02	00.03	00.01	NS	NS	0.044	NS	0.051
C6DC	00.14	00.05	00.02	00.01	00.18	00.06	00.12	00.04	NS	NS	NS	NS	0.075
C6	00.09	00.03	00.12	00.03	00.12	00.02	00.10	00.03	NS	NS	0.008	0.011	NS
C8	00.08	00.02	00.11	00.03	00.12	00.02	00.11	00.03	NS	NS	0.010	0.010	NS
C10:1	00.05	00.01	00.07	00.02	00.07	00.02	00.07	00.02	0.031	NS	0.031	0.004	NS
C10	00.05	00.01	00.08	00.02	00.08	00.01	00.07	00.02	NS	NS	0.003	0.002	NS
C14	00.30	00.08	00.29	00.06	00.36	00.05	00.26	00.08	NS	0.016	NS	NS	0.006
C16	01.05	00.02	01.05	00.02	01.08	00.04	01.04	00.04	NS	0.039	NS	NS	0.011
C18:2	00.04	00.01	00.04	00.01	00.06	00.02	00.05	00.02	0.005	NS	0.033	NS	0.044
C18	00.44	00.08	00.47	00.07	00.53	00.09	00.04	00.01	NS	NS	0.035	NS	0.036
C18OH	00.05	00.02	00.05	00.01	00.08	00.02	00.05	00.02	0.011	NS	NS	NS	0.022
3-Hydroxy/Di-carboxy ACCsa	00.43	00.08	00.05	00.01	00.07	00.03	00.04	00.02	NS	NS	0.010	NS	0.011
Medium-chain ACCsb	0,1896	00.05	0,264	00.09	0,272	00.06	0,24	00.09	NS	NS	0.003	0.002	NS
Long-chain ACCsc	02.06	00.05	02.06	00.03	03.03	00.06	02.05	00.08	NS	NS	0.028	NS	0.008
Val	116	21	124	36	134	25	99	27	NS	NS	0.022	NS	0.014
Leu/Ile/Pro-OH	197	49	205	76	239	55	165	46	NS	NS	0.034	NS	0.013
Tyr	79	18	73	26	128	27	74	24	0.002	<0.001	0.003	NS	<0.001
His	143	54	181	39	184	10	156	32	NS	NS	0.013	0.026	NS

[a]Sum of C4OH/C3DC, C5DC/C6OH, C6DC, and C18OH whole blood concentrations (μmol L^{-1});
[b]Sum of C6, C8, C10:1, and C10 whole blood concentrations (μmol L^{-1}).
[c]Sum of C14, C16, C18:2, and C18 whole blood concentrations (μmol L^{-1}).
