# Supplementary material for: Effects of caloric restriction on neuropathic pain, peripheral nerve degeneration and inflammation in normometabolic and autophagy defective prediabetic Ambra1 mice
Source: PLoS One. 2018 Dec 10;13(12):e0208596. doi: 10.1371/journal.pone.0208596 (PMC6287902; doi:10.1371/journal.pone.0208596)
Supplement: S6 Table — Tables a) and b) show only the significant decrease/increase levels of cytokines analyzed both in nerves tissue lysates samples and in blood. Data are shown as FOLD CHANGE (CCI (ST)/NAIVE or CCI CR/CCI ST). Any ≥ 1. 5-fold increase or ≤ 0. 65-fold decrease in signal intensity for a single analyte between samples may be considered a measurable and significant difference in expression. (PDF) [file pone.0208596.s012.pdf]

**a) Nerve Lysate WT naïve vs WT CCI D7 ST**		
**Mediator**	**Function**	**Fold change**
Eotaxin-1 (CCL11)	chemotactic for eosinophils, basophils, implicated in allergic responses	1,94
Granulocyte colony-stimulating factor (GCSF)	stimulates growth of progenitors of mono, neutro, eosino and baso; activates macrophages	1,95
IL1-beta	induces IL-1,6,8, TNF, GM-CSF by macrophages; proinflammatory	1,90
IL2	promotes the differentiation of immature T cells into regulatory T cells; pro-inflammatory	1,59
IL6	pro-inflammatory cytokine; anti-inflammatory myokine;secreted by T cells and macrophages	2,22
metallopeptidase inhibitor 1 (TIMP-1)	tissue inhibitor of metalloproteinases	4,71
sTNF RI	endogenous inhibitors of TNF	2,61
Eotaxin-1 (CCL11)	chemotactic for eosinophils, basophils, implicated in allergic responses	1,94
**b) Serum WT naïve vs WT CCI D7 ST**		
**Mediator**	**Function**	**Fold change**
CD30LG	cell membrane protein of the tumor necrosis factor receptor family	1,80
Eotaxin-1 (CCL11)	chemotactic for eosinophils, basophils, implicated in allergic responses	2,65
Eotaxin-2 (CCL24)	chemotactic for basophils, Th2 lymphocytes, and tryptase-chymase mast cells	3,46
Granulocyte colony-stimulating factor (GCSF)	stimulates growth of neutro progenitors	2,48
IL-1 beta	induces IL-1,6,8,TNF, GM-CSF by macrophages; proinflammatory	-1,76
IL-6	pro-inflammatory cytokine; anti-inflammatory myokine;secreted by T cells and macrophages	-2,10
IL-12 p40/p70	inflammatory cytokine inducer	-1,86
Keratinocyte-derived chemokine (KC)/chemokine (C-X-C motif) ligand 1	recruits and activates leukocytes	-1,77
TNF alpha	inflammation; induces cytokine secretion; activates macrophages	-1,56
sTNF R I	endogenous inhibitors of TNF	-1,53
sTNF R II	endogenous inhibitors of TNF	-1,86
MIP-1 alpha (CCL3)	chemotactic for monocytes/macrophages, T lymphocytes, basophils and eosinophils	-2,11
